# Bridging the gender, climate, and health gap: the road to COP29

**DOI:** 10.1016/S2542-5196(24)00270-5

**Published:** 2024-11-11

**Authors:** Kim Robin van Daalen, Laura Jung, Sara Dada, Razan Othman, Alanna Barrios-Ruiz, Grace Zurielle Malolos, Kai-Ti Wu, Ana Garza-Salas, Salma El-Gamal, Tarek Ezzine, Parnian Khorsand, Arthur Wyns, Blanca Paniello-Castillo, Sophie Gepp, Maisoon Chowdhury, Ander Santamarta Zamorano, Jess Beagley, Clare Oliver-Williams, Ramit Debnath, Ronita Bardhan, Nicole de Paula, Alexandra Phelan, Rachel Lowe

**Affiliations:** a Barcelona Supercomputing Center, Barcelona, Spain; b British Heart Foundation Cardiovascular Epidemiology Unit, Department of Public Health and Primary Care, University of Cambridge, Cambridge, UK; c Heart and Lung Research Institute, University of Cambridge, Cambridge, UK; d Cambridge Collective Intelligence and Design Group and climaTRACES Lab, University of Cambridge, Cambridge, UK; e Sustainable Design Group, Department of Architecture, University of Cambridge, Cambridge, UK; f Cambridge Public Health, University of Cambridge, Cambridge, UK; g Division of Infectious Diseases and Tropical Medicine, Leipzig University Medical Center, Leipzig, Germany; h University College Dublin Centre for Interdisciplinary Research, Education and Innovation in Health Systems, School of Nursing, Midwifery and Health Systems, University College Dublin, Dublin, Ireland; i The National Ribat University, Khartoum, Sudan; j ISGlobal, Barcelona, Spain; k Escuela de Medicina y Ciencias de la Salud, Tecnológico de Monterrey, Monterrey, Mexico; l College of Medicine, University of the Philippines Manila, Manila, Philippines; m European Citizen Science Association, Berlin, Germany; n Department of Geography, Faculty of Mathematics and Natural Science, Humboldt University of Berlin, Germany; o International Labour Organization, Geneva, Switzerland; p Faculty of Medicine of Tunis, University of Tunis El Manar, Tunis, Tunisia; q Period Futures, Wish for Wash, Atlanta, GA, USA; r University of Melbourne, Melbourne, VIC, Australia; s Universitat Pompeu Fabra (UPF), Barcelona, Spain; t Centre for Planetary Health Policy, Berlin, Germany; u Research Department 2, Potsdam Institute for Climate Impact Research, Member of the Leibniz Association, Potsdam, Germany; v Women in Global Health, Washington, DC, USA; w The Coombe Women and Infants University Hospital, Dublin, Ireland; x Global Climate and Health Alliance, San Francisco, CA, USA; y Public Health Specialty Training Programme, Cambridge, UK; z Caltech-Cambridge Climate and Social Intelligence Lab, California Institute of Technology, Pasadena, CA, USA; aa Machine Intelligence Unit, Indian Statistical Institute, Kolkata, India; ab Women Leaders for Planetary Health, Berlin, Germany; ac Food and Agriculture Organization of the United Nations, Rome, Italy; ad Department of Environmental Health and Engineering, Johns Hopkins University, Baltimore, MD, USA; ae Center for Health Security, Johns Hopkins University, Baltimore, MD, USA; af Centre on Climate Change and Planetary Health and Centre for Mathematical Modelling of Infectious Diseases, London School of Hygiene & Tropical Medicine, London, UK; ag Catalan Institution for Research and Advanced Studies, Barcelona, Spain

## Abstract

Focusing specifically on the gender–climate–health nexus, this Personal View builds on existing feminist works and analyses to discuss why intersectional approaches to climate policy and inclusive representation in climate decision making are crucial for achieving just and equitable solutions to address the impacts of climate change on human health and societies. This Personal View highlights how women, girls, and gender-diverse people often face disproportionate climate-related health impacts, particularly those who experience compounding and overlapping vulnerabilities due to current and former systems of oppression. We summarise the insufficient meaningful inclusion of gender, health, and their intersection in international climate governance. Despite the tendency to conflate gender equality with number-based representation, climate governance under the UNFCCC (1995–2023) remains dominated by men, with several countries projected to take over a decade to achieve gender parity in their Party delegations. Advancing gender-responsiveness in climate policy and implementation and promoting equitable participation in climate governance will not only improve the inclusivity and effectiveness of national strategies, but will also build more resilient, equitable, and healthier societies.

## Introduction

As the world prepares for the 29th UN Conference of the Parties (COP29) Climate Summit in Baku, Azerbaijan, concerns about gender representation and equality have reignited following the initial appointment of 28 men and no women to the COP29 organising committee in January, 2024.^1^ This decision has drawn sharp criticism, with the Women and Gender Constituency, alongside fellow Observer constituencies and over 180 civil society organisations, demanding that gender equality be centred in the COP29 Presidency and its outcomes.^2^

Both slow-onset environmental changes (eg, sea level rise, increasing temperatures, droughts) and sudden-onset events induced by climatic changes (eg, heavy rains, storms, floods) exacerbate systemic inequalities and disproportionately affect marginalised populations, particularly those living in low-income areas.^3^ The populations most affected by climate change tend to be those least responsible for greenhouse gas emissions and are often not sufficiently recognised or prioritised in global climate governance.^4^, ^5^ Although local contexts might vary and intersect with other social characteristics (eg, class, race, ability, sexuality, age, geographical location), women, girls, and gender minorities are often particularly at risk of climate-related adverse impacts.^6^, ^7^ These adverse impacts include threats to their health, an area traditionally neglected in the climate change discourse.^8^, ^9^

Despite decades of efforts to integrate gender considerations within the UN Framework Convention on Climate Change (UNFCCC), progress has been slow.^10^, ^11^, ^12^, ^13^ Feminist scholars have consistently noted that emphasis remains mainly on achieving a gender balance in climate governance, rather than exploring themes such as gender responsiveness, gender equality, or equitable and clean technology transfer.^10^, ^11^, ^12^, ^13^ The UNFCCC insufficiently recognises the role of climate change in worsening gendered health impacts, including gender-based violence and the safeguarding of reproductive and maternal health in the context of climate change.^11^, ^14^ Moreover, although the need for gender balance is often highlighted, climate change governance continues to be dominated by the disproportionate representation of (cisgender) men,^15^ risking the exclusion of diverse needs and perspectives and undermining effective decision making.^16^

In this Personal View, we focus specifically on the gender–climate–health nexus and build on existing feminist works and analyses^7^, ^10^, ^11^, ^17^, ^18^, ^19^, ^20^ to discuss why intersectional approaches to climate policy and inclusive representation in climate decision making are crucial for achieving just and equitable solutions to address the impacts of climate change on human health and societies. This work is contextualised by: reviewing some of the main reported gendered health impacts of climate change; summarising the main decisions and initiatives on gender, health, and their intersection in the history of the UNFCCC; and quantitatively assessing the gender gap in Party delegations at COPs from 1995 (COP1) to 2023 (COP28) and the estimated timelines to achieve gender parity in these delegations.

## The gendered health impacts of climate change

Gender and sex interact as modifiers of major causes of death and morbidity (panel 1).^24^ Biological sex affects physiological responses and disease progression through genetic, epigenetic, and hormonal pathways, whereas (societal) gender roles and norms influence exposure to intermediary determinants of health, such as material or structural (eg, access to resources, health-care utilisation, housing), psychosocial (eg, stressors, relationships), and behavioural factors.^24^, ^26^ An example is the increased morbidity from sexually transmitted infections in women engaging in heterosexual intercourse. Biological factors, such as increased tissue trauma during intercourse, retention of infected ejaculate, and the composition of cervical mucus, increases the likelihood of men-to-women transmission of sexually transmitted infections compared with women-to-men transmission.^27^ From a gender perspective, restrictive sociocultural norms around sexual behaviour can limit women's sexual freedom, access to information, and ability to engage in safe sexual practices, further compounding their health risks.^27^ Such complex sex–gender relationships interact with broader societal, economic, and cultural factors shaping an individual's susceptibility to disease,^28^ including health risks related to climate change.^8^, ^29^, ^30^

Panel 1 **Definition of sex and gender**

In this Personal View, we follow a combination of WHO,^21^ UNICEF,^22^ and the National Institutes of Health Office of Research on Women's Health^23^ definitions of sex and gender.

**Sex**

Sex is defined as the multidimensional biological and physiological characteristics into which humans and most other species are classified based on their reproductive functions, chromosomes, hormones, gene expression, gonads, external genitalia, and secondary sex characteristics. Most individuals fall into two categories: female (eg, XX chromosomes, higher oestrogen and progesterone, reproductive organs, including a vagina, ovaries, and uterus, production of egg cells) or male (eg, XY chromosomes, higher testosterone, reproductive organs, including a penis and testicles, production of sperm). Intersex is an umbrella term used to describe individuals who are born with natural variations in biological or physiological characteristics (including sexual anatomy, reproductive organs, chromosomal patterns, or a combination of these) that do not correspond to a single sex. In many cultures, sex is typically assigned at birth on visual inspection of external genitalia by medical professionals and parents, which often force intersex individuals in a binary sex selection. Intersex individuals face unique medical and social vulnerabilities, as well as stigmatisation and discrimination.^22^, ^23^, ^24^, ^25^

**Gender**

Gender is defined as a multidimensional construct that encompasses gender identity and expression (eg, appearance and behaviour), as well as social and cultural expectations associated with an individual's sex traits (eg, expectations around status, characteristics, and behaviour). As a social construct, gender varies from society to society and can change over time. In many cultures, gender is conceptualised as binary (eg, woman or man), but gender also includes categories, such as non-binary (an umbrella term for identities outside the gender binary), gender fluid (individuals who do not identify with a fixed gender), or Two-Spirit people in some Indigenous communities. Transgender refers to a person whose gender identity is different from the sex they were assigned at birth; cisgender refers to those whose gender identity corresponds to the sex that they were assigned at birth.^22^, ^23^, ^24^, ^25^

**Note on the use of gendered terms in this Personal View**

We acknowledge the limitations of this Personal View in capturing the full spectrum of gender and sex diversity by predominantly using colloquial binary terms, such as “women” and “men” or “female” and “male” to broadly highlight the contrasts and differences that arise on the climate–health nexus due to gender and sex. Although we made efforts to include discussions on gender diverse and sex diverse people, data constraints, societal framing, and scarce scientific evidence often restrict the discussion to these binary categories of gender and sex. Given that gender diverse and sex diverse people are often among the most marginalised in society, more research is needed to understand the impact of climate change on all groups and ensure that diverse perspectives are included in climate governance. We emphasise that gender is non-binary, socially produced, self-identified, and a socioculturally complex phenomenon. Likewise, we underscore that sex is not strictly binary, and encompasses various biological characteristics, such as chromosomes, hormones, and secondary sexual characteristics.

**Figure 1:**
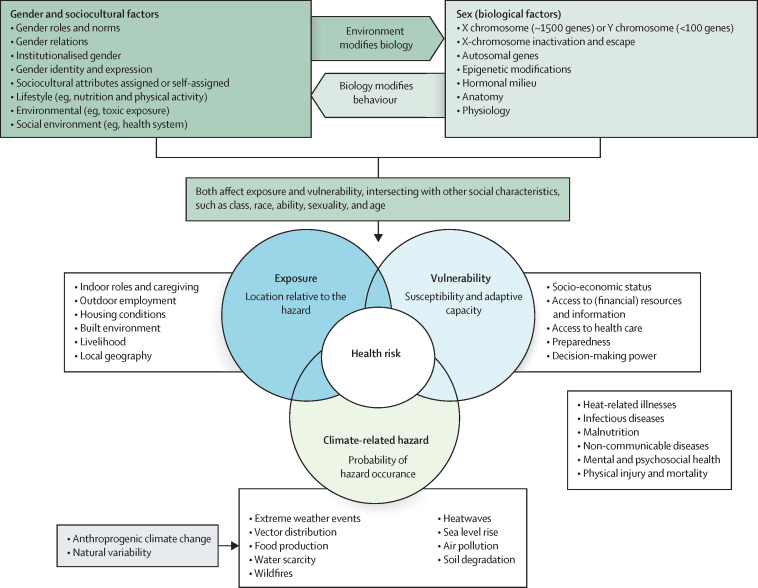
Gender, climate change, and health impacts The type and magnitude of health risks (eg, heat-related illnesses, infectious diseases, malnutrition) from climate change result from a combination of differences in climate hazards (eg, type, probability of occurrence, magnitude), exposure (location relative to the hazard), and vulnerability (susceptibility and adaptive capacity). The sex–gender interaction influences exposure and vulnerability to health risks from climate hazards combined with the context of other social characteristics, such as wealth, class, freedom of expression, level of emancipation, ethnicity, race, and age. The influence of sex–gender on the exposure and vulnerability to climate-related health risks can vary by country or area due to a range of socioeconomic variables and differing gender roles. For many health risks, the knowledge and evidence base of how biological sex, gender, and other sociocultural factors modify climate-related disease phenotypes is still limited. Evidence is particularly scarce when concepts of gender and sex might have opposite effects on the vulnerability and exposure to climate-related disease risks. Men are more likely to work outdoors due to social factors, exposing them to high temperatures for extended periods; whereas, physiologically, some studies suggest that during exercise the core temperature of women increases more in hot and dry climates, with the opposite being true in hot and humid climates.

Women, girls, and gender minorities tend to have a higher climate change burden due to their heightened exposure, vulnerability, and more limited adaptive capacity compared with men and boys—although in some contexts, the inverse applies (figure 1).^29^, ^31^ Vulnerability to climate changes becomes gendered not due to the intrinsic characteristics of women as individuals or as a group, but rather due to the social organisation of women's labour in socioeconomic and cultural circumstances that shape the associated risk factors.^12^ In many countries, compared with men, women are less likely to own land and resources to protect them in post-disaster situations, have less control over income, less access to information, and experience limited institutional support and restricted freedom of association, resulting in increased vulnerability to acute and long-term climate change impacts.^32^ In addition to gendered vulnerabilities to climate impacts, there are gender-differentiated effects of climate mitigation and adaptation approaches. For example, some scholars have argued that poorly designed carbon taxes might disproportionately burden women by not addressing gendered income disparities or not accounting for women's socioeconomic position.^12^, ^33^ Furthermore, in resource-constrained settings, women might not have the agency to effectively adapt to climate change,^34^, ^35^ and are commonly regarded as so-called shock bearers, who are expected to carry out daily tasks (eg, collecting water from distant sources) despite harsh climate conditions.^34^, ^35^ At a large scale, climate mitigation initiatives, such as renewable energy sources from solar arrays or wind turbines, often demand extensive land use rights that are governed by gender norms in many countries. If the existing gendered power imbalances in land access and resource allocation are not tackled, these new energy systems risk further perpetuating the same existing structural inequalities.^36^, ^37^

Although the exact interaction between biological sex, gender, and other sociocultural factors in shaping climate-related health outcomes is still not fully understood, gendered social structures appear to have a substantial role in driving many of the gender disparities in climate-related health risks (panel 2).^8^, ^9^, ^29^, ^30^ For instance, studies in The Gambia and the USA have linked heat stress to adverse birth outcomes, such as reduced fetal strain or spontaneous preterm births,^105^, ^106^ whereas a global umbrella review found that these same adverse pregnancy outcomes were associated with air pollution.^107^ Moreover, climate change exacerbates social stressors contributing to gender-based violence, which amplifies related health risks, such as physical injury, mental health conditions, or exposure to sexually transmitted infections.^100^, ^101^ A recent UN Populations Fund (UNFPA) study projects that gender-based violence against women in sub-Saharan Africa could triple by 2060 if climate change continues under a so-called business-as-usual approach.^108^ Extreme events, which are expected to become more frequent and intense than previously due to climate change, could also severely affect women's social, physical, and mental wellbeing.^88^ For instance, numerous studies highlight that after climate disasters, women's caregiving responsibilities substantially intensify and they are often required to provide emotional support to their families while neglecting their own needs.^88^, ^109^ The increased risk of gender-based violence for women and gender minorities staying in temporary shelters, particularly when accessing sanitation facilities at night, often results in heightened stress and anxiety.^96^, ^97^, ^100^ Furthermore, gender diverse people face stigmatisation and discrimination in many communities, creating increased barriers to accessible and good quality health services and basic needs, particularly during the aftermath of extreme events.^110^

Panel 2 **Overview of the pathways of gender-specific health outcomes impacted by climate change**

**Access to essential health care and services**

• During and after climate-related extreme events, women often have additional challenges in accessing health-care services, particularly for reproductive and maternal health. Critical needs, such as prenatal and postpartum care, safe childbirth, paediatric services, access to contraceptives and menstrual hygiene products, and treatments for menopause symptoms, are frequently deprioritised, exacerbating women's vulnerability during these times.^38^, ^39^
• Before climate-related extreme events, access to information also plays a crucial role. Women can face barriers to accessing information regarding early warning systems or weather alerts or safety protocols. This limited access can hinder their ability to follow appropriate safety plans and secure necessary services during emergencies, increasing their vulnerability.^8^
• Concerns about personal safety or the unavailability of public transportation can make it difficult for women to travel to health-care facilities or cooling centres during extreme heat events, especially if these places are far from home or in unsafe areas.^29^
• Some groups, including pregnant women, are unable to access drugs due to their exclusion from clinical trials (eg, malaria prophylaxis), thus increasing their vulnerability.^40^

**Air pollution and quality**

Many activities that drive climate change, such as burning fossil fuels and agricultural production, contribute to air pollution. Climate-driven changes in weather conditions can also increase ground-level ozone and particulate matter concentrations from drought dust and wildfire smoke.^41^

• Women and children have higher exposure to indoor air pollutants from cooking with polluting fuels and technologies and gathering firewood, increasing their risk of respiratory and health problems.^42^
• The toxicity of the work environment for firefighters, who are predominantly men in many societies, has been exacerbated by escalating climate change, particularly in the wildland or wildland-urban interface.^43^
• Exposure to air pollution during pregnancy can impact fetal and infant growth and development, leading to health outcomes, such as preterm births, low birthweight,^44^, ^45^ or impaired psychomotor development.^46^, ^47^, ^48^
• High concentrations of air pollutants, in particular nitrogen oxides, are associated with bone damage or loss in individuals who are postmenopausal.^49^

**Disaster-related injury and mortality**

The intensity, frequency, duration, timing, and spatial extent of extreme weather and climatic events are changing due to climate change.^50^

• Natural disasters lower the life expectancy of women more than of men, with a higher effect on women from lower socioeconomic status and those living in gender-unequal societies.^51^
• During flooding events, some studies report that men might be at higher risk of drowning events than women, due to higher risk-taking behaviour. Other studies suggest that women are at higher risk, due to the wearing of traditional clothing or being less likely to know how to swim or climb trees.^52^

**Food insecurity and malnutrition**

• Climate change affects food security by reducing agricultural productivity, impacting food availability, access, and nutritional quality, and exacerbating malnutrition and health issues.^53^ Of the 690 million chronically hungry people, 60% are women and girls.^54^
• Women, particularly women-headed households, often have less access to nutrition information and resources compared with men, making them more susceptible to food shortages, food insecurity, and malnutrition.^55^
• In many regions, cultural norms often prioritise men's nutritional needs, making women more likely to skip meals when food access and quality are constrained.^56^, ^57^
• Women are more frequently affected by nutritional deficiencies and anaemia due to varying nutritional needs across different life stages and biological cycles.^30^, ^58^
• Undernutrition during pregnancy can increase the risk of preterm births, stillbirths, and intrauterine growth restriction. Conversely, a healthy pregnancy and breastfeeding during childhood can reduce the risk of chronic diseases later in life.^59^, ^60^
• A combination of rising temperatures, heatwaves, droughts, and rainfall variability can affect water security and impede access to drinking water, resulting in water scarcity and dehydration. Similarly to undernutrition, dehydration during pregnancy can affect fetal growth, stimulate the release of labour-inducing hormones, cause preterm births, and increase maternal risk of anaemia and pre-eclampsia.^61^

**Heat and high temperatures**

High temperatures affect health through both exposure (eg, occupation) and vulnerability (eg, age, comorbidities), leading to multiple health effects, such as increased mortality and heat-related illnesses.^62^, ^63^

• Increased average temperatures and increasing frequency of extreme heat events have been linked to a greater risk of preterm birth, miscarriage,^64^, ^65^, ^66^ congenital anomalies,^67^ gestational hypertension, and pre-eclampsia.^68^ During pregnancy, exposure to heat during crucial stages of brain development can have lasting effects on a child's white matter microstructure.^69^
• Heat-related illnesses refer to incapacitating conditions directly related to a rise in body temperature (eg, heatstroke, exhaustion, syncope, cramps). Systematic reviews report a reduced overall observed rate of heat-related illness in women compared with men at a population level.^62^, ^70^
• Although there is no full consensus, some of the available literature report higher temperature-related all-cause mortality in women compared with men, as well as cardiovascular and respiratory adverse health outcomes.^71^, ^72^, ^73^, ^74^, ^75^
• Temperature physiology studies suggest decreased heat dissipation in women at very high exercise workloads or high levels of heat stress.^76^ Other studies suggest that a women's core temperature increases less in hot and humid environments, whereas the opposite was true in hot and dry climates, and that the thermoregulatory response in women might vary over the menstrual cycle.^77^

**Infectious diseases**

• Climate change can influence the spread and emergence of infectious diseases.^78^ Factors such as community norms and behaviours related to human–animal–environment interactions, access to health care, anatomical and immunological differences, and pregnancy can lead to varying outcomes of climate-sensitive infectious diseases among genders.^79^
• In some regions, women and girls have a higher risk of contracting vector-borne diseases because their traditional roles often involve collecting and storing water, as well as spending more time around artificial mosquito breeding habitats around the house, increasing their exposure to mosquitoes.^30^ However, regional differences have a role and, in some contexts, men bear a higher risk for infections, such as schistosomiasis, due to their greater occupational exposure to water bodies.^80^
• Men are more commonly at risk for leptospirosis due to activities such as animal caretaking, farming, fishing, and playing water sports.^81^, ^82^ Women in rural communities often care for backyard chickens and ducks,^83^ making them more susceptible to avian influenza. This increased vulnerability is due to generally low education levels and low access to information and training on avian influenza.^81^, ^84^
• Pregnant women are more susceptible to several climate-sensitive vector-borne diseases (eg, Zika, dengue, malaria) due to physiological, behavioural, and social vulnerabilities. These infections can result in various adverse pregnancy outcomes and congenital abnormalities (eg, congenital Zika syndrome).^46^, ^85^

**Mental health and wellbeing**

As climate change drives the increasing frequency and severity of climate-related hazards, mental health and psychosocial issues are also being affected, resulting in trauma, emotional distress, the emergence of new conditions, and the worsening of pre-existing ones.^86^, ^87^

• Women appear to be more susceptible to the development of climate-related anxiety, major depressive disorder, worry, and stress compared with men.^88^, ^89^, ^90^
• In extreme weather events or climate disasters, women often experience higher burdens of care and responsibility, which further exacerbates and negatively impacts mental health.^88^
• Men might be at higher risk of suicide and severe depression in the face of drought and resulting agricultural losses in some regions.^8^

**Migration and displacement**

• Climate-induced environmental change drives human migration.^91^ According to UN data, 80% of people displaced by climate change are women.^92^
• As climate-related hazards devastate existing livelihoods, women risk being left behind, burdened with the responsibility of taking care of households, whereas men migrate in search of better opportunities abroad, oftentimes to financially support the family. Additionally, sociocultural norms often restrict women's mobility, access to transportation, and freedom of movement, which further limits their ability to adapt to disasters.^93^, ^94^, ^95^
• Women experience anxiety and stress from temporary shelters not meeting their needs (eg, breastfeeding, menstruation) and experience increased vulnerability to attack or abuse in temporary shelters, for example when accessing water, sanitation, and hygiene facilities at night.^96^, ^97^

**Sexual and reproductive health and rights**

Access to sexual and reproductive health services are hindered in climate change emergencies, including access to contraception, HIV treatment and prophylaxis, and abortion services. Supply chains for commodities are also disrupted and health facilities can be damaged or inaccessible.^98^

• Sexual and reproductive health services might have scarce resources and are often deprioritised to respond to immediate needs in crises.^99^
• Extreme weather events and climate disasters might also disrupt access to menstrual hygiene products and sanitation facilities.^96^
• During extreme weather events, barriers to safe sexual protective strategies and compromised access to healthcare (eg, prevention, screening treatment) contribute to increased rates of sexually transmitted infections, HIV, and unwanted pregnancies. In regions where access to abortion is limited, the paucity of protective strategies for safe sex can be particularly risky.^98^

**Violence**

• Several forms of gender-based violence towards women and girls have been reported to increase during or after extreme events (eg, heatwaves, droughts, floods, storms, wildfires), including sexual violence and harassment, physical violence, so-called witch killings, early or forced marriage, and emotional violence. These incidents are often linked to factors, such as economic instability, disrupted infrastructure, food or water insecurity, and an increase in distance travelled or mental distress, which exacerbate social inequities in the aftermath of such extreme events.^100^, ^101^
• Climate change and environmental degradation heighten the risks of violence against women and girls, driven by factors such as displacement, financial stressors and resource scarcity, or disruptions in services.^101^

**Housing and built environment**

• Poorly built environments can restrict ventilation and cooling indoors, as well as outdoor retention and increased absorption of heat (ie, heat island effects). In many societies, as temperatures rise and the intensity of heatwaves increases, women who spend more time indoors are at a higher risk of mental and physical health issues related to poor-quality homes.^102^
• Women in low-income communities often do not have a say in household energy decisions, resulting in maladaptations during extreme weather events. In male-led households, women might avoid using cooling devices when alone, even during extreme heat.^103^
• Access to outdoor cooling infrastructure, such as cooling towers, green space, and shade, are often gendered. In some settings, women tend to refrain from using such public infrastructure due to gendered cultural hierarchy and general safety concerns.^104^
• The impact of sex and gender on health outcomes related to climate change or its drivers might vary by country and area due to a range of socioeconomic variables and differing gender roles in local settings. This panel provides examples of gender-specific health impacts associated with climate change and its drivers, but it is not meant to be a comprehensive or exhaustive overview or universally generalisable or applicable to all contexts.

Note: the impact of sex and gender on health outcomes related to climate change or its drivers might vary by country and area due to a range of socioeconomic variables and differing gender roles in local settings. This panel provides examples of gender-specific health impacts associated with climate change and its drivers, but it is not meant to be a comprehensive or exhaustive overview or universally generalisable or applicable to all contexts.

Individuals are situated within broader political, economic, and sociocultural relations and intersectional privileges. Sex–gender considerations in climate-related health research and policy need to be placed within the context of other factors influencing exposure and vulnerability including, but not limited to, wealth, class, freedom of expression, sexuality, ethnicity, race, religiosity, and age.^7^, ^28^ This intersectionality should be particularly emphasised because marginalised groups, such as low-income households, migrants and displaced people, and Indigenous peoples, are structurally at higher risk of climate-related adverse impacts than non-marginalised groups.^4^, ^28^

## Integration of gender and health in international climate policy

**Figure 2:**
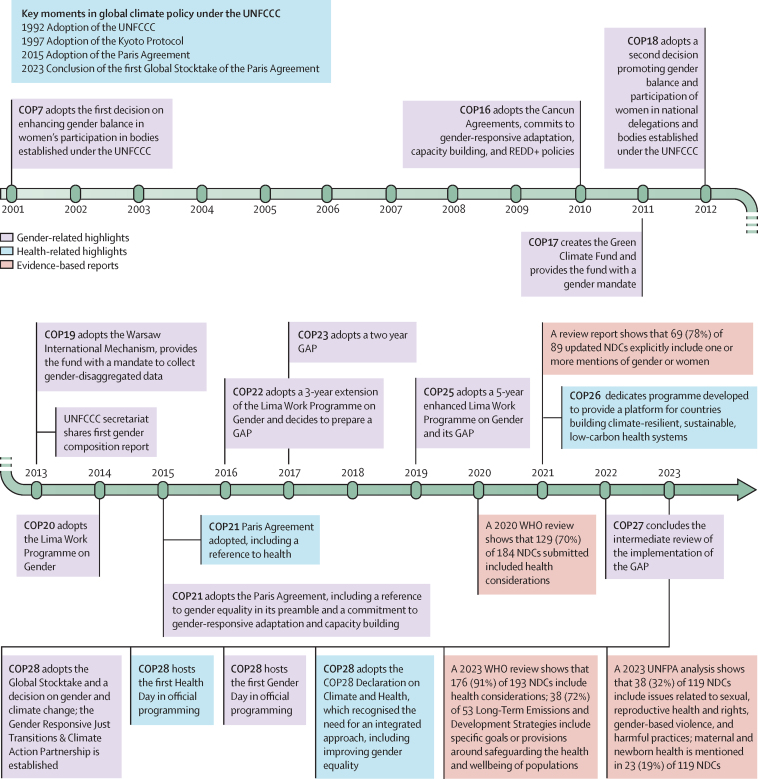
Summary timeline of key decisions and initiatives on health and gender in the UNFCCC (1992–2023)^111^, ^112^, ^113^, ^114^ NDCs=Nationally Determined Contributions. REDD+=reducing emissions from deforestation and forest degradation in developing countries plus additional forest-related activities that protect the climate (ie, sustainable management of forests and the conservation and enhancement of forest carbon stocks). GAP=Gender Action Plan. UNFCCC=UN Framework Convention on Climate Change.

Climate governance and legally binding obligations have been negotiated within the context of the UNFCCC since its adoption in 1992, including key international treaties, such as the Kyoto Protocol in 1997 and the Paris Agreement in 2015. The UNFCCC initially predominantly focused on mitigation (ie, reducing, avoiding, and stabilising greenhouse gases in the atmosphere) and included a narrow set of voices; however, discussions around adaptation (ie, avoiding harm to ecological, social, or economic systems by adapting to climate change) and loss and damage (ie, climate change consequences that cannot be avoided by adaptation) have been increasingly elevated, and more diverse voices have been included.^11^ Recognition of gender, as well as health, has slowly progressed, but remains limited in climate negotiations (figure 2).^11^, ^115^, ^116^

A pivotal moment for the recognition of gender occurred in COP7 in 2001, marking the first formal decision to enhance women's participation in climate decision making (figure 2).^11^ However, robust mechanisms to enhance women's participation were not introduced until 2009. Such mechanisms include the establishment of the Women and Gender Constituency and the launch of the Women's Delegate Fund,^117^, ^118^ which have played crucial roles in advocating for gender-responsive and gender-transformative approaches in climate action and supporting women's participation in climate governance.^11^ Subsequent multilateral decisions further institutionalised gender within the UNFCCC, leading to more structured and ongoing efforts to integrate gender into climate governance.^11^ Examples include the Cancun Agreements at COP16 and the Lima Work Programme on Gender (LWPG) adopted at COP20, alongside the Gender Action Plan (GAP), which was first accepted at COP23. The GAP sets out priorities under five thematic areas to advance knowledge and understanding of gender-responsive climate action. Overall, the Paris Agreement, adopted at COP21, acknowledges gender equality and women's empowerment, particularly in adaptation and capacity building. Yet, countries have only taken up these commitments to a limited extent in their Nationally Determined Contributions (NDCs), which tend to focus on gender balance over substantive gender-responsive policies and actions.^119^

Most recently, at COP28, the Gender-Responsive Just Transition and Climate Action Partnership was launched. Endorsed by 60 Parties, the partnership includes a package of commitments surrounding data, financing, and equal opportunities to ensure climate action and the transition to a low-carbon economy are gender responsive.^120^ Parties further agreed to review the LWPG and the GAP to address progress, challenges, gaps, and priorities in implementing the GAP by COP29.^121^ The first global stocktake at COP28, a formal assessment of the progress towards mitigating global warming since the Paris Agreement, renewed encouragement for gender-responsive policies, with several key organisations (eg, Green Climate Fund) committing to mainstream gender.^122^ Despite these advancements, current gender considerations seem to remain largely focused on climate adaptation strategies to date, neglecting the crucial gendered impacts of mitigation efforts, the loss and damage agenda, and climate financing.^11^, ^116^

From the health perspective, there is also slow progress (figure 2).^115^ Despite the human right to health being recognised in the Paris Agreement in 2015, it was not until 2017 that health gained policy relevance in the COP space: when the COP23 Presidency tasked WHO with producing the landmark COP24 special report on climate and health.^123^ Shortly after, the COP26 Health Programme was launched in Glasgow, an initiative for building health systems that are sustainable low-carbon, climate-resilient, or both. Building on this momentum, the Alliance on Transformative Action on Climate and Health was soon established as a platform to support the implementation of these commitments, to which 84 countries are committed as of October, 2024.^124^

More concrete outcomes could be observed during COP28, with the first climate-health ministerial convened, the endorsement of the COP28 Declaration on Climate and Health by 151 countries (as of October, 2024), the first official health day, the highest number of climate–health side-events, and funding commitments of over US$1 billion dedicated to climate and health.^115^ Assessments of NDCs and their updates show increasing attention to health considerations.^111^, ^112^, ^125^ However, most NDCs do not include national climate–health assessments, set or monitor explicit health targets, or integrate health within a wider context of social determinants.^111^, ^112^, ^125^

Despite the substantial gendered health impacts of climate change described earlier and their recognition by other UN bodies, notably WHO,^112^, ^126^ UNFPA,^113^ and the Commission on the Status of Women,^127^ gendered climate-related health impacts are insufficiently considered in official global climate governance mechanisms.^11^ For example, a 2023 UNFPA analysis found that only 38 (32%) of 119 NDCs addressed issues related to sexual and reproductive health and gender-based violence, and only 23 (19%) mentioned maternal and newborn health.^113^ The LWPG and GAP do not include substantive content on sexual and reproductive health and rights, nor do they mention health more broadly. Although Finland and Sweden raised concerns about the need for an integrated approach that incorporates gender equality in climate-related initiatives in the annex, the COP28 declaration on climate and health makes only a single reference to women and girls, limited to policy development and implementation, with no mention of broader gender considerations. Furthermore, the non-binding nature of the agreement and the absence of accountability mechanisms underscore the continued ambiguity in achieving tangible outcomes.^128^ Coordination across civil society groups is also necessary to promote integrated responses to climate, gender, and health. Seeking to support collaboration between sexual and reproductive health and rights and climate-focused organisations, the Sexual and Reproductive Health and Rights and Climate Justice Coalition was established in 2021 and works to mobilise collective strengths to advance gender equality and reproductive health in the context of climate change.^129^

## The ongoing gender imbalance

**Figure 3:**
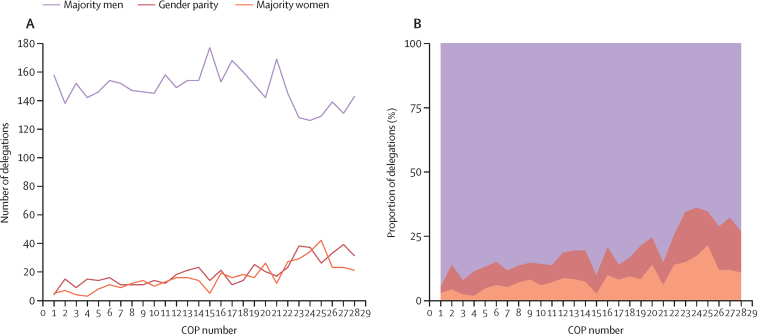
Inferred delegation composition from COP1 to COP28 (1994–2023) Majority men is defined as >55% inferred men; gender parity is defined as 45–55% inferred women and gender minorities; majority women is defined as >55% inferred women and gender minorities. (A) Number of delegations with majority men, gender parity, or majority women. (B) Proportion (%) of delegations with majority men, gender parity, or majority women. COP=Conference of the Parties.

**Figure 4:**
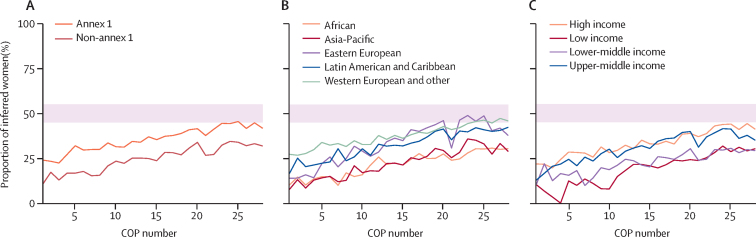
Proportion of inferred women from COP1 to COP28 (1995–2023) (A) Proportion (%) of inferred women in Parties' delegation by type of party to the Convention. Annex-1 Parties are those that are legally bound to reduce greenhouse gas emissions under the Kyoto protocol; non-annex 1 Parties are those that are only required to report greenhouse gas emissions. (B) Proportion (%) of inferred women in Parties' delegation by UN region. (C) Proportion (%) of inferred women in Parties' delegation by World Bank income group. The pink shading represents gender parity, which is defined as 45–55% women or gender minorities. COP=Conference of the Parties.

Regardless of the range of multilaterally adopted goals to improve gender participation in UNFCCC bodies^130^, ^131^, ^132^ and the general domination of language around gender balance over more meaningful inclusion of gender (equality) within the UNFCCC,^11^, ^116^ trends in the gender composition of Party delegations to the COPs show that women and gender minority voices remain under-represented (figure 3; figure 4).

Supporting the observations and work of previous organisations, scholars, and activists,^15^, ^132^ we analysed gender representation among representatives of Party and Observer State delegations at COPs between 1995 and 2023. A full description of the methods can be found in the appendix, as well as details on the overall summary characteristics of the collected data (appendix p 21). Briefly, after manually extracting delegates' information from (final) UNFCCC participant lists, gender was inferred based on gendered prefixes (eg, Mr, Ms, Mme, Sr, Sra, Dato, Datin), gendered language (eg, he, she, they, her, him, them) in publicly available online documentation, or a gender-to-name-algorithm (for 266 [0·2%] of 149 010 delegates).^115^, ^133^, ^134^ As only a few people (two of 149 010 delegates) were inferred as non-binary or gender non-confirming, and given the limitations of these analyses in accurately inferring gender outside the binary (appendix p 4), gender minorities were included in the categorisation of “women” for the purpose of these analyses and discussion.

Our analyses on 149 010 Party delegates representing 5152 delegations (appendix p 21) show that during 1995–2023, only 543 (10·5%) of 5152 delegations displayed gender parity (ie, delegations contained 45–55% women and gender minorities), whereas 4156 (80·7%) of delegations were composed of majority men and 453 (8·8%) of delegations of majority women (figure 3). A slight positive trend in the percentage of inferred women delegates could be observed across different UN regions, income groups, and type of party to the Convention over 1995–2023 (figure 4). However, in 2023 at COP28, 143 (73%) of 195 Party delegations were still majority men, and only 31 (16%) of 195 showed gender parity (figure 3).

**Figure 5:**
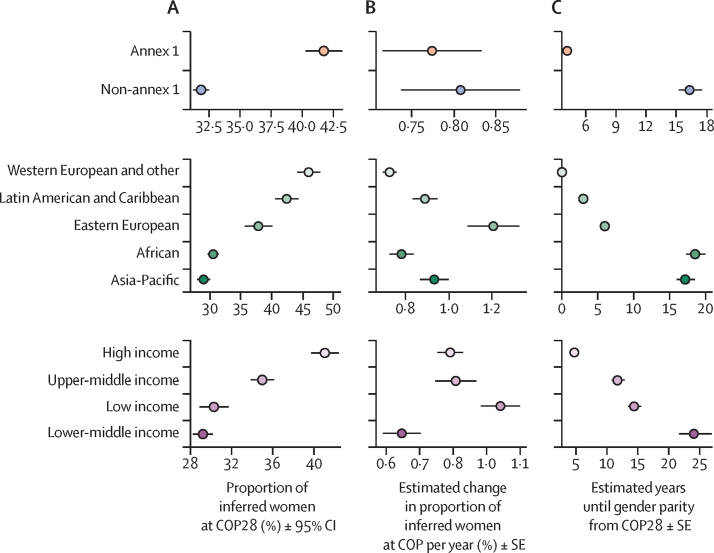
Inferred women representation from COP1 to COP28 (1994–2023) (A) Proportion of inferred women at COP28. (B) Estimated change in proportion of inferred women at COP per year. (C) Estimated years until gender parity from COP28. Data are presented grouped by Party to the Convention (Top), UN regional grouping (middle), or Worldbank income group (bottom). Gender parity is defined as 45–55% inferred women and gender minorities. COP=Conference of the Parties.

When assessing regional groupings, our data reveal that gender parity has not yet been achieved in most UN regions, except for the “Western European and Other” category (figure 4B). Based on current trends in the percentage increase of women's representation per year, it will be at least a decade from COP28 before the Asia–Pacific and Africa regions reach parity in COP delegations (figure 5). A similar result is found comparing the annex I grouping (ie, Parties legally bound to reduce greenhouse gas emissions under the Kyoto protocol) with the non-annex I grouping (ie, Parties that are only required to report greenhouse gas emissions; figure 5). When assessing representation across World Bank income groups, a widening gap in gender representation between the high-income category and the low-income category was found, with an estimate of around 24 years to reach gender parity in the lower-middle income category (figure 5).

**Figure 6:**
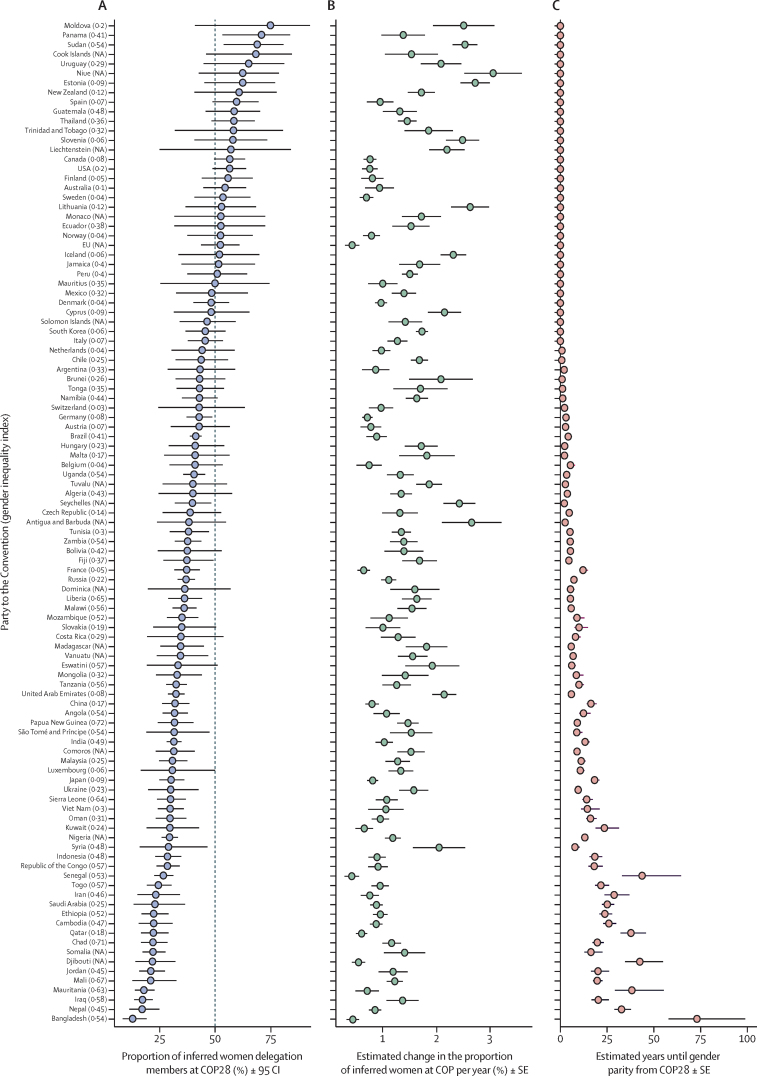
Women's representation in Party delegations (A) Proportion (%) of inferred women delegation members at the COP28 (2023). (B) Estimated change of inferred women delegation members at the COP per year. (C) Estimated years until gender parity (ie, 45–55% inferred women and gender minorities) from COP28 (2023). Only countries or territories that were a Party or Observer to the Convention in COP28 were included. Data are presented for those Party or Observer states with a trend in estimated change of percentage of women delegation members per year, adjusted p-value <0·01. The gender inequality index of each Party for 2019 is depicted between brackets. COP=Conference of the Parties.

Women's representation in COP28, percent change in women's representation (1995–2023), and years until gender parity is reached from COP28 varies widely across Parties (figure 6; appendix pp 23, 24). When comparing Parties with adjusted p-values of less than 0·01 for trend, only one Party, Bangladesh, was estimated to take over 50 years to reach gender parity, whereas others are expected to take several years (figure 6). 34 Parties were considered to have already reached parity (ie, percentage women at COP28 >45%; figure 6). More variation can be observed when comparing Parties with adjusted p-values for trends of greater than 0·01 and less than 0·05 (appendix pp 23, 24).

**Figure 7:**
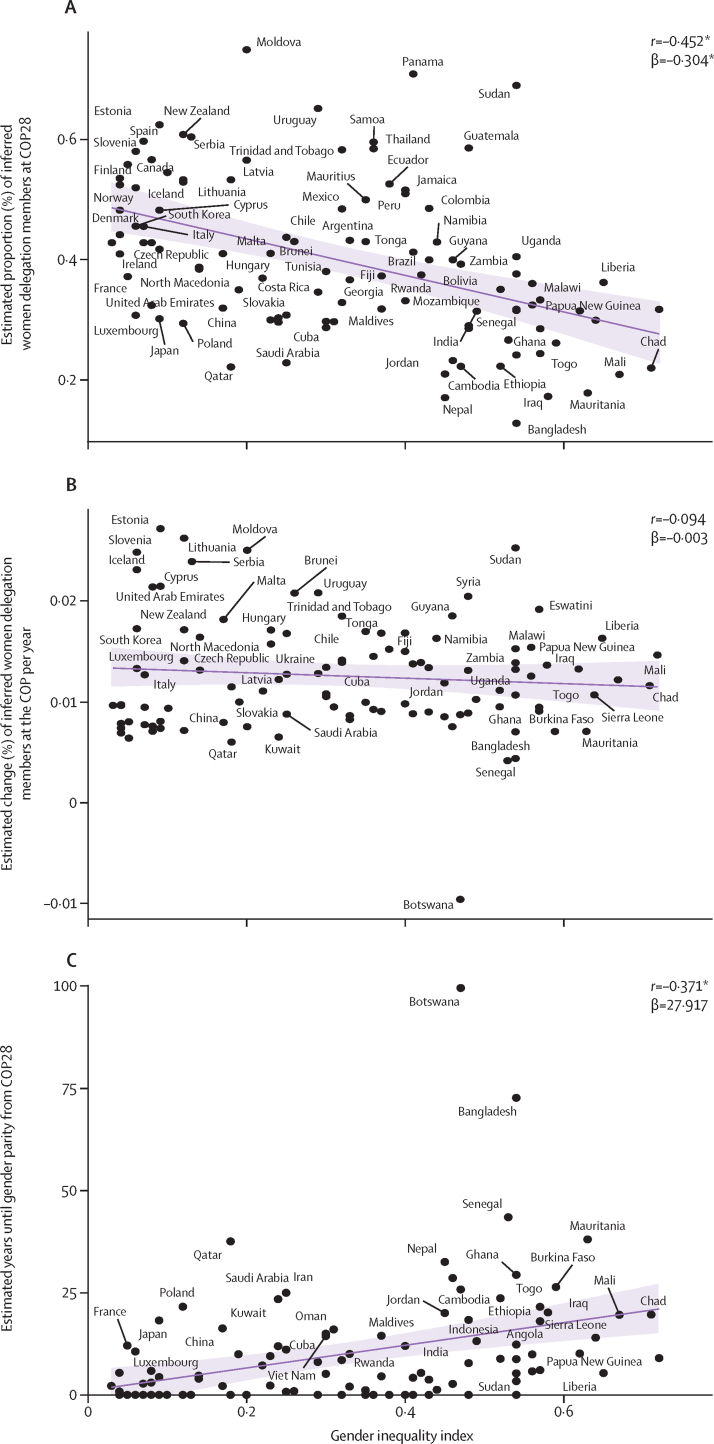
Women representation by Party gender inequality index (A) Proportion (%) of inferred women delegation members at the COP28 (2023) by their 2019 gender inequality indexes. (B) Estimated change (%) of inferred women delegation members at the COP per year by their 2019 gender inequality indexes. (C) Estimated years until gender parity (ie, 45–55% inferred women and gender minorities) from COP28 (2023) by their 2019 gender inequality indexes. Only countries with a trend in estimated change of percentage of women delegation members per year reported, adjusted p-value <0·05 were included. The purple shaded areas represents the 95% confidence interval of the trendline. *p<0·001. COP=Conference of the Parties.

We explored women's representation in COP28, percent change in women's representation at previous COPs (1995–2023), and number of years until gender parity is reached from COP28, and how these relate to the gender inequality index (GII; a composite metric of gender inequality). We found negative trends when plotting women's representation in COP28 and percent change in women's representation per year against GII, and a positive trend when plotting years until gender parity is reached from COP28 against GII. In other words, less gender-equal countries tend to have fewer women on their Party delegations, have a lower rate of change towards gender parity, and are more likely to take more years to reach gender parity (figure 7).

## The need for equitable representation

Insufficient representation is a symptom of a broken societal system whereby governance is not inclusive. Inadequate representation risks excluding unique expertise, perspectives, and lived realities and reinforces inequitable power structures. Beyond the imperative of representative justice,^135^ more equitable inclusion of women has consistently been suggested in theory-driven and empirical work to transform policy making across political and social systems.^136^, ^137^ Transforming policy making includes the generation of policies that better present women's interests.^136^, ^137^ Evidence on the effects of more inclusive representation has been especially strong in the field of public health,^138^, ^139^, ^140^, ^141^ which is closely intertwined with environmental factors,^142^ as not only do healthy environments benefit the population, but tackling public health issues, such as air pollution and clean water access, also has positive effects on the environment.

For instance, recent analyses of 49 European countries revealed that greater women's political representation correlates with reduced inequalities in self-reported health, lower geographical inequalities in infant mortality, and fewer disability-adjusted life-years lost across genders.^143^ Similarly, a study of 155 countries found large negative associations between women's political representation and mortality, especially in weak democratic contexts.^138^ Corresponding findings are reported at both local and national levels. For example, after accounting for endogeneity, increased political representation in Indian state legislatures was associated with a drop in neonatal mortality, as women leaders were more likely to support health facilities, antenatal care coverage, and immunisations.^144^ Even the introduction of quotas has affected legislative attention, particularly regarding issues related to public health (eg, children's and women's health), poverty alleviation, and women's rights, despite heterogeneity (eg, in experiences, identities, roles) among women within and across different geographical and cultural settings.^136^, ^139^, ^145^

Similar positive findings related to environmental policies have been reported, with women's representation in national parliaments being associated with increased ratification of environmental treaties^146^ and more stringent climate change policies.^147^ Furthermore, women legislators in the European parliament and US House of Representatives are more inclined to support environmental legislation than men.^148^, ^149^ Although unclear through which mechanisms, some analyses suggest a distinct cross-sectional pattern, with nations where women have higher political status tending to have lower CO_2_ emissions, when controlling for other factors.^150^ At the local level, including women in forest and fishery management groups has resulted in better resource governance and conservation outcomes in south Asian countries.^151^, ^152^ A randomised field experiment further supports these findings, revealing that gender quotas among local forest users from Indonesia, Peru, and Tanzania resulted in more effective climate interventions. Specifically, groups with gender quotas conserved more trees and shared payments more equally.^153^

These findings suggest a common thread: achieving more equitable gender representation is not just a matter of fairness, but a strategic imperative. Equitable representation has the potential to drive the development of more comprehensive and effective policies that address a broader spectrum of societal needs, including those relevant to public and environmental health.^136^, ^137^, ^138^, ^139^, ^140^ Therefore, ensuring gender equity in representation might be essential for enabling societies to more effectively confront and resolve these complex challenges.

## Going beyond the gender balance

However, although our quantitative analyses focused on achieving gender balance, merely including a critical mass of women does not necessarily, by itself, guarantee the development and implementation of more gender-responsive or more effective climate policy making.^136^, ^154^, ^155^ Women are not inherently feminist, nor are men inherently impeding gender-responsive policies.^156^ In contrast with some of the previously cited studies,^146^, ^147^, ^148^, ^149^, ^150^, ^151^, ^152^, ^153^ some research on women's involvement in climate governance suggest that increased representation does not always lead to meaningful policy changes.^12^, ^154^ Illustratively, a study on descriptive representation in Scandinavian countries found that more representation did not lead to explicit substantive change in the recognition of gender-specific approaches in climate strategies.^154^ Some participants reasoned that this is because gender equality was “already realised”, despite scholars noting evidence of gender differences in material conditions and attitudes relevant to Scandinavian climate policy.^154^ Using post-structural feminist international relations theory, the authors argue their findings might have been due to masculine norms being deeply institutionalised in climate institutions, shaping how policy makers adapt and operate.^154^

Moreover, even when formally included, women's active participation in male-dominated institutions is often constrained by existing social norms, implicit biases, and structural barriers.^10^, ^145^, ^157^ For example, the Indian and Nepali REDD+ projects, which aimed to have equal numbers of women and men in decision making, reported that women had little influence on the decision making processes, and were not listened to.^158^, ^159^ The net positive effects of quotas, as a means to improve gender balance, are not always universal,^136^ tend not to reflect women's intersectionality with other groups (eg, race, class), and often favour women who are comparatively powerful already.^11^, ^160^ Quotas can even result in reinforcing or exacerbating gendered power structures, or assigning so-called women's issues or gender issues to women.^13^, ^145^, ^161^ Furthermore, previous research highlights how the terms of engagement under UNFCCC negotiating spaces allow for a narrow view on gender and are disadvantageous for those who do not represent Global North interests, and do not have the capital, connections, or other resources to challenge these interests.^10^ An absence of intersectionality within UNFCCC debates results in the conceptualisation of gender within mostly White western perspectives.^10^

As other scholars have emphasised,^11^, ^12^, ^13^ solely focusing on the so-called numbers game is unlikely to produce substantially better or more equitable gender outcomes without also addressing the gendered barriers to full inclusion in governance, and without the recognition that women are not a homogenous group.^11^, ^12^, ^13^ A narrow focus on balancing numbers in representation risks diverting attention from feminist-driven reforms that aim to dismantle systems of oppression—including the patriarchy, colonialism, and capitalism—that are causative of climate change.^11^, ^12^, ^13^ Overall, although improving the gender balance in governance is important as part of the democratic process, this is only a small step towards meaningfully integrating gender and equity into climate policy and its implementation.

Gender affects how people and their wellbeing are affected by and able to adapt to the changing climate*.* Meaningfully incorporating gender into climate policy and practice means identifying and recognising gender-specific risks and vulnerabilities (ie, gender sensitive), and addressing them and their causes (ie, gender responsive) through all phases of programme and policy development.^162^, ^163^ Meaningful gender incorporation requires investment in the enabling conditions (ie, inclusive governance structures, climate financing and gender-responsive budgeting, international development partnerships, active civil society, gender analysis and evidence, and enabling cultural practices) that ensure effectiveness, efficiency, and long-term sustainability of these actions.^164^ Throughout, it is crucial to recognise the diversity of women and their embodiment of multiple, intersecting identities that shape their climate experiences and mitigation and adaptation needs. Reducing women to a single, homogenous group not only risks deepening existing inequalities, but also overlooks opportunities to address the varied needs of all individuals.^7^, ^13^ Crucially, addressing these issues requires a clear recognition of the unique gendered challenges to human health and safeguarding sexual and reproductive health and rights in the context of a changing climate.^29^, ^30^, ^96^, ^100^ Yet, feminist scholars^10^, ^11^, ^12^, ^13^ consistently report that gender remains neglected in international, national, and local climate governance, and where gender is considered, positive action is not always assured.^10^, ^11^, ^12^, ^13^ For example, an absence of proper financing and monitoring can hinder progress,^10^, ^11^, ^12^, ^13^ or strategies based on flawed gender assumptions (eg, women are innately caring and connected to the environment, women are homogeneously vulnerable, gender equality is a women's issue, and gender equality is about numbers) risk having unintended or counterproductive consequences.^13^ However, the full extent of the meaningful incorporation of gender equality is difficult to assess, as there tends to be a paucity of evaluation on the implementation of climate policies.

## Recognising the unique position of gender and sex minorities

Gender diverse individuals are widely recognised to have unique health-related and climate-related risks due to their increased vulnerability, stigma, and discrimination.^165^, ^166^, ^167^ Illustratively, during and after extreme events, transgender people in the USA report being threatened or prohibited access to shelters.^168^ Similarly, in the Philippines, Indonesia, and Samoa, gender diverse individuals, such as *bakla, waria*, and *Fa'afafin*e—biologically male people who regularly perform roles traditionally associated with women—often face discrimination, mockery, and exclusion from evacuation centres or access to food.^167^ There remain major gaps in knowledge about the health implication of climate change for gender-diverse people. Organisational initiatives aimed at addressing gender disparities focus predominantly on cisgender women and girls, and the few practice and policy recommendations that include gender-diverse people only focus on disaster relief.^169^ More gender-sensitive data on the climate-related health effects for gender diverse people is urgently needed to inform policy and practice.

Although we made efforts to include discussions on gender diverse people, data constraints, societal framing, and scarce scientific evidence often restrict the discussion beyond these binary categories of gender and sex. Therefore, we acknowledge the limitations of this Personal View in capturing the full spectrum of gender and sex diversity by predominantly using colloquial binary terms, such as “women” and “men” or “female” and “male” to broadly highlight the contrasts and differences that arise on the climate–health nexus due to gender.

## The road to COP29

In this Personal View, we focus on the gender–climate–health nexus to underscore the crucial need for intersectional gender integration into climate policy as both a matter of equity and a strategic necessity for effective, sustainable outcomes for all people and their wellbeing. We emphasise how women, girls, and gender diverse people often have disproportionate climate-related health impacts, particularly those who experience compounding and overlapping vulnerabilities due current and former systems of oppression. Additionally, we summarise the limited meaningful inclusion of gender, health, and their intersection in international climate governance, and show that despite the tendency to conflate gender equality with numbers-based representation, climate governance under the UNFCCC remains dominated by men, with several countries projected to take over a decade to achieve gender parity in their Party delegations.

With countries preparing to submit their third round of NDCs in early 2025, this is a crucial opportunity for proactivity. The gender dimensions of climate change and its associated vulnerabilities, impacts, adaptive capacity, and interventions—including sexual and reproductive health and rights, gender-based violence, and other gendered health issues—as well as gender responsiveness should be strengthened and integrated into NDCs, National Adaptation Plans, and other national and local key climate policies. Proactive attunement includes incorporating gender considerations explicitly into substantive and procedural obligations. Although binding obligations will be an important step in ensuring states are accountable, these are often subject to greater levels of scrutiny and negotiation than non-binding obligations, and might take time to be formally adopted. COP29 Parties should therefore also consider adopting obligations, including non-binding substantive and procedural goals, to shape norms and build traction to incorporate gender considerations into international climate law. Gender-responsive commitments should include commitments to collect sex-disaggregated and gender-disaggregated data (beyond the binary) on the varied health-related impacts of climate change across genders, the allocation of resources and investments in the necessary enabling conditions, and addressing the persisting societal gender inequalities in economic, social, and legal life. Although there is potential to elevate health considerations within the UNFCCC's Gender Action Plan, it is crucial to mainstream gender and health issues across all aspects of the climate negotiations, rather than confining them to a single workstream.

By advancing gender-responsiveness in climate action, and fostering equitable participation in governance, countries will not only improve the inclusivity and effectiveness of national strategies, but also help to build more resilient, equitable, and healthier societies better equipped to confront the climate crisis.

### Contributors

## Declaration of interests

KRvD receives funding by the Gates Cambridge Scholarship (OPP1144) for her PhD research. RD acknowledges the support from Cambridge Humanities Research Grant, Keynes Fund, CRASSH Grants (climaTRACES laboratory), and UK Research and Innovation International Science Partnership Funds. RL acknowledges a Royal Society Dorothy Hodgkin Fellowship. KRvD and RL acknowledge funding from the EU Horizon Europe research and innovation programme under grant agreement 101057131 and grant agreement 101057554, which are both part of the EU climate change and health cluster. NdP works for the Food and Agriculture Organization of the UN; and is responsible for views expressed in this publication, which do not necessarily reflect the views or policies of the Food and Agriculture Organization of the UN. BP-C acknowledges funding from the European Union's Horizon 2020 research and innovation programme under grant agreement 865564 (European Research Council Consolidator Grant EARLY-ADAPT) and the Swedish Research Council (FORMAS) under grant agreement 2022-01845 (project ADATES); and acknowledges support from the grant CEX2023-0001290-S funded by MCIN/AEI/10.13039/501100011033, and support from the Generalitat de Catalunya through the CERCA Program. All other authors declare no competing interests.
