# Supplementary material for: Bridging the gender, climate, and health gap: the road to COP29
Source: Lancet Planet Health. 2024 Nov 11;8(12):e1088–105. doi: 10.1016/S2542-5196(24)00270-5 (PMC11634786; doi:10.1016/S2542-5196(24)00270-5)
Supplement: Supplementary appendix [file mmc1.pdf]

THE LANCET
Planetary Health

Supplementary appendix

This appendix formed part of the original submission and has been peer reviewed. We post it as supplied by the authors.

Supplement to: van Daalen KR, Jung L, Dada S, et al. Bridging the gender, climate, and health gap: the road to COP29. *Lancet Planet Health* 2024; published online Nov 11. https://doi.org/10.1016/S2542-5196(24)00270-5.

Supplemental material to "Bridging the gender, climate, and health gap: the road to COP29"

Kim Robin van Daalen *PhD*,[1,2,3] Laura Jung *MD*,[4] Sara Dada *MSc*,[5] Razan Othman *MBBS*,[6,7] Alanna Barrios-Ruiz *MD*,[8] Grace Zurielle Malolos *MD*,[9] Ka-Ti Wu *MSc*,[10,11] Ana Garza-Salas *MD*,[8] Salma Elgamal *MSc*,[12] Tarek Ezzine *BMSc*,[13] Parnian Khorsand *MPH*,[14] Arthur Wyns *MSc*,[15] Blanca Paniello-Castillo *MMSc*,[7,16] Sophie Gepp *MD*,[17] Maisoon Chowdhury *MPH*,[18] Ander Santamarta Zamorano *MPH*,[19] Jess Beagley *MSc*,[20] Clare Oliver-Williams *PhD*,[21] Ramit Debnath *PhD*,[22,23,24] Ronita Bardhan *PhD*,[25,26] Nicole de Paula *PhD*,[27,28] Alexandra Phelan *SJD*,[±29,30] Rachel Lowe *PhD*[±*1,31,32]

[±]These authors contributed equally. [*] Refers to full professors.

Methodology gender representation of COPs

These supplement materials describe the methodology for the section of the Viewpoint discussing the representation of women at international climate change decision-making and are based on the work by van Daalen *et al.* 2022.[1]

Data source and extraction

For the purpose of this analysis, we focused on gender representation at the Conference of the Parties (COPs) and particularly within delegations of Parties to the UNFCCC (or Convention). The Parties to the Convention predominantly include countries, but it also includes representation of the catholic church (i.e., Holy See), former countries (e.g., Yugoslavia, Serbia and Montenegro), and *de jure* sovereign states (i.e., State of Palestine). Other observers, such as representatives of non-governmental organisations (NGOs), and international governmental organisations (IGOs) are excluded.

The official lists of delegates were obtained from the UNFCCC website (https://unfccc.int/documents) for COP1 (1995) through COP28 (2023). A full list of documentation used can be found in **Supplement Table 1**.

Country, prefixes, and (full) names of all Party delegates were manually extracted and collected by thirteen researchers. A second member of the team double-checked all extracted data.

Inferring likely gender

Likely gender (i.e., man, woman, non-binary or gender non-conforming) was primarily inferred based on prefixes (e.g., Mr., Ms., Sr., Sra., M., Mme., Mx.) provided in the obtained lists of delegates (see **Supplement Table 2**) [i.e., for 99.8% of delegates, n=148664]. When the prefix was not available in

the list with delegates, online biographies were utilised to infer gender based on gendered prefixes, gendered pronouns (e.g., he/she/they, her/him/them), or other gendered language (for 0.2% of delegates, n=266). A gender-to-name-algorithm was utilised (https://genderize.io/) when the former two methods were not available (n=80) and only accepted when the probabilistic certainty score was ≥0.50 (for 0.05% of delegates, n=80). This algorithm is based on historical databases combining first name and country. Whilst this tool has been checked for robustness in multiple previous studies, the algorithm cannot infer people outside of the gender binary and has reduced quality for inferring gender for non-Western names.[2–4] Expression or gender presentation (phenotype) from images/photos were not used to infer gender, due to the subjectivity and the likelihood to wrongfully infer someone's gender.

Data cleaning and coding

Countries that underwent name changes in the period assessed (but did not change geographic boundaries) have been re-coded to their current (2024) country name (e.g. Swaziland to Eswatini, Zaire to Democratic Republic of the Congo, Turkey to Türkiye). Countries that changed geographic boundaries and/or geopolitical context were not re-coded (e.g. Yugoslavia, Serbia and Montenegro). **Supplement Table 3** includes an overview of re-coded and not re-coded (former) countries.

Each Party was assigned to their corresponding United Nations (UN) country region (Asia-Pacific States, Eastern European States, African States, Western European and other States, Latin American and Carribean States), Worldbank (WB) income grouping 2022 (high-income, upper-middle-income, lower-middle-income, low-income), and Gender Inequality Index (GII) 2019. The GII is a measure developed by the United Nations Development Programme (UNDP) that measures gender inequalities in three aspects of human development: empowerment, economic status and health. It ranges from 0-1, with lower values corresponding to decreased disparities between the genders.[5] Countries were further categorised into Annex 1 (countries that are legally bound to reduce GHG emissions under the Kyoto protocol) and non-Annex 1 countries (countries that are only required to report GHG emissions).[6] Two Worldwide Governance Indicators (2019) were also included; the Voice and Accountability indicator ("*a reflection of the perceived extent to which a country's citizens are able to participate in selecting their government, freedom of expression, freedom of association and free media*") and Government Effectiveness indicator ("*a reflection of the perceived public services quality, civil service quality and degree of independence from political pressure, policy formulation and implementation quality, and the credibility of government's commitment to policies")*. Estimates of governance performance on these indicators ranges from 2.5 (strong) to -2.5 (weak).[7]

To be able to include (former) countries in longitudinal analyses, they have been grouped following their UN region groupings that they would theoretically fall in based on their geographic location (e.g. Yugoslavia was categorised under "Eastern Europe"). These categorisations can be found in

Supplement Table 4, and are marked in light blue. The WB income group, GII, and Worldwide Governance Indicators were not extended.

Data analysis and visualisation

Inferred gender composition of each delegation was categorised into majority women (>55% women or gender minorities), gender parity (45%–55%) and majority men (>55% men). This was presented as the total number of delegations and the percentage of delegations with majority women, gender parity and majority men over time (1995-2023) [**Figure 2**]. Please note that in our primary analysis, we have intentionally focused on examining gender parity within each Party delegation rather than the overall percentage of women at each COP. This is because large delegations with many women could distort the results and create a more favourable impression of women's representation than is accurate at local and regional levels.

Binomial 95% confidence intervals (CI) were calculated for the proportions of interest (percent of women). To estimate the percent change in women per year ± standard error (SE), we fit a linear regression model. This was then used to estimate the number of years until gender parity was reached using COP28 (2023) as the baseline. The (i) estimated proportion (%) ± 95% CI of inferred women delegation members at the COP28, (ii) estimated change (%) ± SE of inferred women delegation members at the COP per year and (iii) estimated years ± SE until gender parity from COP28 (2023) — were presented by type of Party to the Convention, UN region, WB income group. The *P*-values for trend (β) were adjusted using the false discovery rate (FDR). The former (i, ii and iii) were presented separately for countries that were Parties in COP28 with an FDR adjusted *P*-value for trend <0.01 (**Figure 3**), 0.01>p value<0.05 (**Supplement Figure 2**) and p value>0.05 (**Supplement Figure 3**).

Selecting only the countries with an FDR adjusted *P*-value for trend (β) of <0.05—i, ii and iii were plotted at a country level against the GII (2019) [**Figure 6**], the Voice and Accountability Worldwide Governance Indicator (2019) [**Supplement Figure 4**] and the Government Effectiveness Worldwide Governance Indicator (2019) [**Supplement Figure 5**]. Linear regression models were fitted, and the Pearson's Correlation Coefficient was calculated.

Missing values were excluded from all analyses. All statistical analyses and data visualizations were conducted in R version 4.0.5 (R Foundation, Vienna, Austria, www.r-project.org). For data visualization, the tidyverse, dplyr, pals, and ggplot packages were used.

Ethical Considerations

All data used for this study was not restricted nor sensitive, nor did it require permission to access or collate. Data was publicly available and accessible, eliminating the need for additional ethical approval.

Research team

The research team comprises an internationally diverse group of professional backgrounds (i.e., architectural engineer, biomedical scientist, computer scientist, data scientist, epidemiologist, geographer, lawyer, medical doctor, policy advisor, social scientist) and those in training for these professions (i.e., students) from a wide variety of socio-cultural backgrounds (Belgium, Egypt, Germany, Ireland, India, Mexico, Netherlands, Philippines, Sudan, Spain, Switzerland, Tunisia, United Kingdom, United States) and languages (Arabic, Basque, Bengali, Catalan, Chinese (Mandarin), Dutch, English, Farsi, Filipino, French, German, Hausa, Hindi, Spanish) which allowed the team to include non-English/non-Western sources and perspectives.

Limitations

Firstly, inference of likely gender depended on prefixes provided in UNFCCC documentation (99.8% of all delegates), gendered language used in online bibliographies, and a gender-to-name algorithm - instead of self-identification of delegates. Notably, non-binary prefixes were unavailable or unselectable during most of the period assessed. Consequently, a delegate's choice of gendered prefix may not reflect their true identity, especially for those without the social or legal freedom to express their gender openly (i.e. due to criminalisation or stigmatisation in their country). As a result, inferring gender was limited by a predominantly binary definition of gender and likely led to the misrepresentation of some delegates' true gender identities. Secondly, our analysis does not allow for an assessment of the influence delegates have on the UNFCCC decision-making processes (e.g., decisions may have been made prior to political convenings), nor whether an increase in gender diversity of Parties' delegations leads to more just gender-transformative policy making. Importantly, many decisions made on behalf of Parties during the COP, may have already been agreed upon before the COP convenes. Thirdly, whilst this analysis provides insight into the representation of gender, it was not able to capture other identity characteristics, such as race, ethnicity, disability status or socio-economic background that may intersect in different dynamics of inequality and discrimination.

Supplement Table 1. Overview with documentation used to extract delegation members for each Conference of the Parties (1-28)

COP	Location	Date(s)	Publication date	Documentation reference	Link to source
1	Berlin, Germany	28/03-07/04 1995	06/04/1995	FCCC/1995/INF.5/Rev.2	https://ccsr.aori.u-tokyo.ac.jp/old/unfccc3/records/600000324.html
2	Geneva, Switzerland	08/07-19/07 1996	19/07/1996	FCCC/CP/1996/INF.2	https://unfccc.int/cop3/fccc/listpart/particip.html
3	Kyoto, Japan	1/12-10/12 1997	09/12/1997	FCCC/CP/1997/INF.5	https://digitallibrary.un.org/record/690376?ln=en
4	Buenos Aires, Argentina	02/11-13/11 1998	12/11/1998	FCCC/CP/1998/INF.8	https://ccsr.aori.u-tokyo.ac.jp/old/unfccc1/records/600000798.html
5	Bonn, Germany	25/10-5/11 1999	4/11/1999	FCCC/CP/1999/INF.3	https://unfccc.int/cop5/resource/docs99.html
6	Bonn, Germany	16/07-27/07 2001	26/07/2001	FCCC/CP/2001/INF.2	https://ccsr.aori.u-tokyo.ac.jp/old/unfccc3/records/600001050.html
7	Marrakesh, Morocco	29/10-9/11 2001	08/11/2001	FCCC/CP/2001/INF.4	https://ccsr.aori.u-tokyo.ac.jp/old/unfccc3/records/600001707.html
8	New Delhi, India	23/10-01/11 2002	1/11/2002	FCCC/CP/2002/INF.2	https://ccsr.aori.u-tokyo.ac.jp/old/unfccc3/records/600002189.html
9	Milan, Italy	01/12-12/12 2003	11/12/2003	FCCC/CP/2003/INF.1	https://unfccc.int/documents/3541
10	Buenos Aires, Argentina	06/12-17/12 2004	17/12/2004	FCCC/CP/2004/INF.3	https://unfccc.int/resource/docs/cop10/inf03.pdf
11	Montreal, Canada	28/11-09/12 2005	9/12/2005	FCCC/CP/2005/INF.2 (Part 1)	https://unfccc.int/resource/docs/2005/cop11/eng/inf02p01.pdf
12	Nairobi, Kenya	06/11-17/11 2006	16/12/2006	FCCC/CP/2006/INF.1	https://unfccc.int/resource/docs/2006/cop12/eng/inf01.pdf
13	Bali, Indonesia	03/12-14/12 2007	14/12/2007	FCCC/CP/2007/INF.1 (Part 1)	https://unfccc.int/resource/docs/2007/cop13/eng/inf01p01.pdf
14	Poznan, Poland	01/12-12/12 2008	11/12/2008	FCCC/CP/2008/INF.1 (Part 1)	https://unfccc.int/resource/docs/2008/cop14/eng/inf01p01.pdf

15	Copenhagen, Denmark	07/12-18/12 2009	16/03/2010	FCCC/CP/2009/INF.1(Part 1)<br>FCCC/CP/2009/INF.1(Part 2)	https://unfccc.int/documents/6107<br>https://unfccc.int/documents/6108
16	Cancun, Mexico	29/11-10/12 2010	10/12/2010	FCCC/CP/2010/INF.1 (Part 1)<br>FCCC/CP/2010/INF.1 (Part 2)	https://unfccc.int/documents/6498<br>https://unfccc.int/documents/6499
17	Durban, South Africa	28/11-9/12 2011	08/12/2011	FCCC/CP/2011/INF.3 (Part 1)<br>FCCC/CP/2011/INF.3 (Part 2)	https://unfccc.int/documents/6989
18	Doha, Qatar	26/11-7/12 2012	07/12/2012	FCCC/CP/2012/INF.2	https://unfccc.int/documents/7631
19	Warsaw, Poland	11/11-22/11 2013	21/11/2013	FCCC/CP/2013/INF.4	https://unfccc.int/resource/docs/2013/cop19/eng/inf04.pdf
20	Lima, Peru	01/12-12/12 2014	12/12/2014	FCCC/CP/2014/INF.2	https://unfccc.int/documents/8579
21	Paris, France	30/11-11/12 2015	11/12/2015	FCCC/CP/2015/INF/2 (Part 1)<br>FCCC/CP/2015/INF.3 (Part 2)	https://unfccc.int/documents/8984<br>https://unfccc.int/documents/9059
22	Marrakech, Morocco	07/11-18/11 2016	18/11/2016	FCCC/CP/2016/INF.3 (Part 1)<br>FCCC/CP/2016/INF.3 (Part 2)<br>FCCC/CP/2016/INF.3 (Part 3)	https://unfccc.int/documents/9494<br>https://unfccc.int/documents/9612<br>https://unfccc.int/documents/9613
23	Bonn, Germany	07/11-17/11 2017	17/11/2017	FCCC/CP/2017/INF.4	https://unfccc.int/sites/default/files/resource/inf04_0.pdf
24	Katowice, Poland	02/12-14/12 2018	14/12/2018	FCCC/CP/2018/INF.3	https://documents-dds-ny.un.org/doc/UNDOC/GEN/G18/366/32/PDF/G1836632.pdf?OpenElement
25	Madrid, Spain	02/12-13/12 2019	13/12/2019	FCCC/CP/2019/INF.4	https://unfccc.int/sites/default/files/resource/cp_inf4.pdf
26	Glasgow, UK	31/10-12/11 2021	23/11/2021	FCCC/CP/2021/INF.3 (Part 1)	https://unfccc.int/sites/default/files/resource/cp2021_inf03p01.pdf

27	Sharm el-Sheikh, Egypt	06/11-18/11 2022	02/12/2022	FCCC/CP/2022/INF.3 (Part 1)	https://unfccc.int/documents/624508
28	Dubai, United Arab Emirates	30/11-13/12 2023	22/12/2023	FCCC/CP/2023/INF.3	https://unfccc.int/documents/636676

Supplement Table 2. Overview of gendered prefixes used for inferring likely gender based on the list of delegates provided in UNFCCC documentation.

**Men's prefixes**	**Women's Prefixes**	**Non-binary prefixes**	**Not gendered prefixes**
Dato	Datin	Mx.	Captain (Capt.)
Datuk	Her Majesty		Dr.
His Majesty	Mme.		H.E.
Lord	Mrs.		Prof.
M.	Ms.		Mx.
Mr.	Princess		
Msgr.	Queen		
Prince	Soeur (sister)		
Sr.	Sra.		
Tan Sri	Srta.		

Supplement Table 3. Re-coding of (former) countries and their geopolitical context.

**Recoded (former country names)**		
**Extracted as**	**Re-coded as**	**Geopolitical context**
Swaziland	Eswatini	Swaziland was the English name for the Kingdom of Eswatini. Swaziland was a British protectorate from 1903-1968. The name was officially changed to Eswatini (the Swazi language) in 2018.
Zaire	Democratic Republic of the Congo	Following a coup in 1965, the country was renamed as the Republic of Zaire in 1971 until 1997 when its name reverted to the Democratic Republic of the Congo.
Libyan Arab Jamahirya	Libya	The fall of the last pro-Gaddafi site and Gaddafi's assassination in 2011 marked the end of the Libyan Arab Jamahirya.
Former Yugoslav Republic of Macedonia	Republic of North Macedonia	In 1991, the Republic of Macedonia (which was renamed as the Republic of North Macedonia in 2019) became one of the successor states of Yugoslavia.
Turkey	Türkiye	Following a formal request of the Turkish authorities Turkey has been officially recognised as Türkiye in international organisations since 2022.

**Not recoded, including (former) countries**	
Holy See	The Holy See is the universal government of the Catholic Church. It operates from the Vatican City State which is a sovereign, independent territory. The Holy See has a permanent observer status at the United Nations.
Palestine	Palestine is considered to be a de jure sovereign state in Western Asia, comprising the Gaza strip, West Bank and parts of modern Israel.
Serbia and Montenegro	Serbia and Montenegro existed between 1992-2006, when it was dissolved after the breakup of Yugoslavia.
Yugoslavia	A nation that was founded after WWI and was dissolved after a number of conflicts in the early 1990s. Was made up of what is present day Bosnia and Herzegovina, Croatia, Macedonia, Montenegro, Serbia (including the regions of Kosovo and Vojvodina) and Slovenia.

Supplement Table 4. Party and Observer State groupings.

Country	Type Convention	UN Regional Group	WB Income Group	GII 2019 value	GII Rank	Per capita GHG	GHG rank	WB_VA	WB_GE
Afghanistan	Non-Annex 1	Asia-Pacific States	Low-income	0.655	157	0.2	178	-1.08	-1.52
Albania	Non-Annex 1	Eastern European States	Upper-middle income	0.181	42	1.94	112	0.9	-0.14
Algeria	Non-Annex 1	African States	Lower-middle income	0.429	103	3.59	81	-1.1	-0.53
Andorra	Non-Annex 1	Western European and other States	High-income	NA	NA	5.97	48	1.09	1.83
Angola	Non-Annex 1	African States	Lower-middle income	0.536	132	0.89	139	0.81	-1.18
Antigua and Barbuda	Non-Annex 1	Latin American and Caribbean States	High-income	NA	NA	5.50	53	0.74	-0.15
Argentina	Non-Annex 1	Latin American and Caribbean States	Upper-middle income	0.328	75	3.99	72	0.59	-0.22
Armenia	Non-Annex 1	Eastern European States	Upper-middle income	0.245	54	1.88	114	0.04	-0.12
Australia	Annex 1	Western European and other States	High-income	0.097	25	15.48	8	1.3	1.62
Austria	Annex 1	Western European and other States	High-income	0.069	14	7.15	33	1.4	1.66
Azerbaijan	Non-Annex 1	Eastern European States	Upper-middle income	0.323	73	3.22	87	-1.55	-0.17
Bahamas	Non-Annex 1	Latin American and Caribbean States	High-income	0.341	77	5.86	49	0.92	0.45
Bahrain	Non-Annex 1	Asia-Pacific States	High-income	0.212	49	19.59	4	-1.46	0.43
Bangladesh	Non-Annex 1	Asia-Pacific States	Lower-middle income	0.537	133	0.51	157	-0.77	-0.79
Barbados	Non-Annex 1	Latin American and Caribbean States	High-income	0.252	56	4.36	66	1.13	0.49
Belarus	Annex 1	Eastern European States	Upper-middle income	0.118	31	6.28	43	-1.46	-0.73
Belgium	Annex 1	Western European and other States	High-income	0.043	4	8.18	26	1.28	1.12

Belize	Non-Annex 1	Latin American and Caribbean States	Lower-middle income	0.415	97	1.78	119	0.53	-0.65
Benin	Non-Annex 1	African States	Lower-middle income	0.612	148	0.69	147	-0.08	-0.26
Bhutan	Non-Annex 1	Asia-Pacific States	Lower-middle income	0.421	99	1.83	117	0.15	0.37
Bolivia	Non-Annex 1	Latin American and Caribbean States	Lower-middle income	0.417	98	2.00	111	-0.07	-0.56
Bosnia and Herzegovina	Non-Annex 1	Eastern European States	Upper-middle income	0.149	38	6.78	36	-0.32	-0.98
Botswana	Non-Annex 1	African States	Upper-middle income	0.465	116	3.64	79	0.49	0.26
Brazil	Non-Annex 1	Latin American and Caribbean States	Upper-middle income	0.408	95	2.04	110	0.26	-0.45
Brunei Darussalam	Non-Annex 1	Asia-Pacific States	High-income	0.255	60	16.64	5	-0.93	1.44
Bulgaria	Annex 1	Eastern European States	Upper-middle income	0.206	48	5.85	50	0.26	-0.07
Burkina Faso	Non-Annex 1	African States	Low-income	0.594	147	0.22	176	-0.17	-0.67
Burundi	Non-Annex 1	African States	Low-income	0.504	124	0.05	190	-1.54	-1.26
Cambodia	Non-Annex 1	Asia-Pacific States	Lower-middle income	0.474	117	0.69	148	-1.36	-0.42
Cameroon	Non-Annex 1	African States	Lower-middle income	0.56	141	0.34	165	-1.21	-0.88
Canada	Annex 1	Western European and other States	High-income	0.08	19	15.50	7	1.48	1.64
Cape Verde	Non-Annex 1	African States	Lower-middle income	0.397	89	1.14	131	0.92	0.25
Central African Republic	Non-Annex 1	African States	Low-income	0.68	159	0.07	188	-1.27	-1.69
Chad	Non-Annex 1	African States	Low-income	0.71	160	0.07	189	-1.42	-1.46
Chile	Non-Annex 1	Latin American and Caribbean States	High-income	0.247	55	4.62	63	1.02	0.99
China	Non-Annex 1	Asia-Pacific States	Upper-middle income	0.168	39	7.35	32	-1.65	0.65
Colombia	Non-Annex 1	Latin American and Caribbean States	Upper-middle income	0.428	101	1.60	126	0.15	0.04

Comoros	Non-Annex 1	African States	Lower-middle income	NA	NA	0.31	167	-0.76	-1.59
Cook Islands	Non-Annex 1	Asia-Pacific States	NA	NA	NA	NA	NA	0.92	NA
Costa Rica	Non-Annex 1	Latin American and Caribbean States	Upper-middle income	0.288	62	1.65	123	1.14	0.36
Côte d'Ivoire	Non-Annex 1	African States	Lower-middle income	0.638	153	0.40	163	-0.48	-0.48
Croatia	Annex 1	Eastern European States	High-income	0.116	29	4.06	69	0.58	0.44
Cuba	Non-Annex 1	Latin American and Caribbean States	Upper-middle income	0.304	67	2.20	105	-1.42	-0.17
Cyprus	Annex 1	Asia-Pacific States	High-income	0.086	21	6.08	46	0.91	0.88
Czech Republic	Annex 1	Eastern European States	High-income	0.136	36	9.64	19	0.98	0.96
Democratic Republic of the Congo	Non-Annex 1	African States	Low-income	0.617	150	0.03	192	-1.28	-1.69
Denmark	Annex 1	Western European and other States	High-income	0.038	2	5.76	51	1.52	1.89
Djibouti	Non-Annex 1	African States	Lower-middle income	NA	NA	0.51	158	-1.42	-0.68
Dominica	Non-Annex 1	Latin American and Caribbean States	Upper-middle income	NA	NA	2.51	99	0.88	-0.18
Dominican Republic	Non-Annex 1	Latin American and Caribbean States	Upper-middle income	0.455	112	2.36	103	0.22	-0.33
Ecuador	Non-Annex 1	Latin American and Caribbean States	Upper-middle income	0.384	86	2.31	104	0.02	-0.44
Egypt	Non-Annex 1	African States	Lower-middle income	0.449	108	2.50	100	-1.49	-0.55
El Salvador	Non-Annex 1	Latin American and Caribbean States	Lower-middle income	0.383	85	1.06	133	0.04	-0.36
Equatorial Guinea	Non-Annex 1	African States	Upper-middle income	NA	NA	5.10	56	-1.83	-1.47
Eritrea	Non-Annex 1	African States	Low-income	NA	NA	0.23	174	-2.08	-1.62
Estonia	Annex 1	Eastern European States	High-income	0.086	21	12.10	16	1.17	1.34
Eswatini	Non-Annex 1	African States	Lower-middle income	0.567	143	0.96	137	-1.33	-0.77

Ethiopia	Non-Annex 1	African States	Low-income	0.517	125	0.15	180	-1.02	-0.55
European Union	Annex 1	Western European and other States	NA	NA	NA	NA	NA	0.03	0.3
Fiji	Non-Annex 1	Asia-Pacific States	Upper-middle income	0.37	84	2.15	108	1.62	1.95
Finland	Annex 1	Western European and other States	High-income	0.047	7	8.04	27	1.07	1.25
France	Annex 1	Western European and other States	High-income	0.049	8	4.62	64	1.29	1.32
Gabon	Non-Annex 1	African States	Upper-middle income	0.525	128	2.18	107	-0.99	-0.91
Gambia	Non-Annex 1	African States	Low-income	0.612	148	0.25	173	-0.2	-0.67
Georgia	Non-Annex 1	Eastern European States	Upper-middle income	0.331	76	2.54	98	0.06	0.79
Germany	Annex 1	Western European and other States	High-income	0.084	20	8.56	23	1.38	1.36
Ghana	Non-Annex 1	African States	Lower-middle income	0.538	135	0.54	156	0.58	-0.15
Greece	Annex 1	Western European and other States	High-income	0.116	29	6.08	45	0.97	0.44
Grenada	Non-Annex 1	Latin American and Caribbean States	Upper-middle income	NA	NA	2.69	94	0.69	-0.07
Guatemala	Non-Annex 1	Latin American and Caribbean States	Upper-middle income	0.479	119	1.11	132	-0.39	-0.69
Guinea	Non-Annex 1	African States	Low-income	NA	NA	0.25	172	-0.92	-0.89
Guinea-Bissau	Non-Annex 1	African States	Low-income	NA	NA	0.17	179	-0.47	-1.43
Guyana	Non-Annex 1	Latin American and Caribbean States	Upper-middle income	0.462	115	3.13	91	0.21	-0.44
Haiti	Non-Annex 1	Latin American and Caribbean States	Lower-middle income	0.636	152	0.30	168	-0.84	-2.03
Holy See	NA	Western European and other States	NA	NA	NA	NA	NA	NA	NA
Honduras	Non-Annex 1	Latin American and Caribbean States	Lower-middle income	0.423	100	1.02	134	-0.6	-0.6
Hungary	Annex 1	Eastern European States	High-income	0.233	51	4.75	62	0.39	0.58

Iceland	Annex 1	Western European and other States	High-income	0.058	9	6.24	44	1.39	1.52
India	Non-Annex 1	Asia-Pacific States	Lower-middle income	0.488	123	1.80	118	0.15	0.39
Indonesia	Non-Annex 1	Asia-Pacific States	Lower-middle income	0.48	121	2.18	106	0.1	0.37
Iran (Islamic Republic of)	Non-Annex 1	Asia-Pacific States	Lower-middle income	0.459	113	7.69	28	-1.48	-0.99
Iraq	Non-Annex 1	Asia-Pacific States	Upper-middle income	0.577	146	4.90	59	-1.01	-1.33
Ireland	Annex 1	Western European and other States	High-income	0.093	23	7.62	29	1.39	1.48
Israel	Non-Annex 1	Western European and other States	High-income	0.109	26	6.98	35	0.66	1.1
Italy	Annex 1	Western European and other States	High-income	0.069	14	5.38	55	1.06	0.4
Jamaica	Non-Annex 1	Latin American and Caribbean States	Upper-middle income	0.396	88	2.90	92	0.63	0.41
Japan	Annex 1	Asia-Pacific States	High-income	0.094	24	8.74	22	0.99	1.6
Jordan	Non-Annex 1	Asia-Pacific States	Upper-middle income	0.45	109	2.48	101	-0.75	0.11
Kazakhstan	Non-Annex 1	Asia-Pacific States	Upper-middle income	0.19	44	12.06	17	-1.19	0.16
Kenya	Non-Annex 1	African States	Lower-middle income	0.518	126	0.36	164	-0.34	-0.35
Kiribati	Non-Annex 1	Asia-Pacific States	Lower-middle income	NA	NA	0.69	146	1.13	-0.14
Kuwait	Non-Annex 1	Asia-Pacific States	High-income	0.242	53	21.62	2	-0.68	-0.16
Kyrgyzstan	Non-Annex 1	Asia-Pacific States	Lower-middle income	0.369	82	1.74	120	-0.59	-0.54
Lao People’s Democratic Republic	Non-Annex 1	Asia-Pacific States	Lower-middle income	0.459	113	2.66	95	-1.8	-0.77
Latvia	Annex 1	Eastern European States	High-income	0.176	41	3.96	73	0.87	0.88
Lebanon	Non-Annex 1	Asia-Pacific States	Upper-middle income	0.411	96	4.04	71	-0.56	-1.17
Lesotho	Non-Annex 1	African States	Lower-middle income	0.553	139	1.22	129	0	-0.91

Liberia	Non-Annex 1	African States	Low-income	0.65	156	0.27	171	-0.08	-1.41
Libya	Non-Annex 1	African States	Upper-middle income	0.252	56	8.83	20	-1.38	-2.01
Liechtenstein	Annex 1	Western European and other States	High-income	NA	NA	3.69	78	1.26	1.82
Lithuania	Annex 1	Eastern European States	High-income	0.124	34	4.14	68	1.01	1.06
Luxembourg	Annex 1	Western European and other States	High-income	0.065	12	15.33	9	1.5	1.84
Madagascar	Non-Annex 1	African States	Low-income	NA	NA	0.13	183	-0.28	-1
Malawi	Non-Annex 1	African States	Low-income	0.565	142	0.09	187	-0.03	-0.79
Malaysia	Non-Annex 1	Asia-Pacific States	Upper-middle income	0.253	59	7.60	30	-0.15	1.04
Maldives	Non-Annex 1	Asia-Pacific States	Upper-middle income	0.369	82	3.70	77	-0.33	-0.11
Mali	Non-Annex 1	African States	Low-income	0.671	158	0.29	169	-0.73	-1.15
Malta	Annex 1	Western European and other States	High-income	0.175	40	3.20	88	1.12	1.04
Marshall Islands	Non-Annex 1	Asia-Pacific States	Upper-middle income	NA	NA	3.25	86	1.14	-1.41
Mauritania	Non-Annex 1	African States	Lower-middle income	0.634	151	0.91	138	-0.84	-0.77
Mauritius	Non-Annex 1	African States	Upper-middle income	0.347	78	3.26	85	0.74	0.87
Mexico	Non-Annex 1	Latin American and Caribbean States	Upper-middle income	0.322	71	3.74	75	-0.04	-0.16
Micronesia (Federated States of)	Non-Annex 1	Asia-Pacific States	Lower-middle income	NA	NA	1.60	127	1.13	-0.08
Moldova	Non-Annex 1	Eastern European States	Upper-middle income	0.204	46	3.17	89	-0.05	-0.46
Monaco	Annex 1	Western European and other States	High-income	NA	NA	NA	NA	0.67	NA
Mongolia	Non-Annex 1	Asia-Pacific States	Lower-middle income	0.322	71	6.73	38	0.38	-0.34
Montenegro	Non-Annex 1	Eastern European States	Upper-middle income	0.109	26	4.05	70	0.04	-0.02

Morocco	Non-Annex 1	African States	Lower-middle income	0.454	111	1.85	115	-0.61	-0.03
Mozambique	Non-Annex 1	African States	Low-income	0.523	127	0.23	175	-0.6	-0.72
Myanmar	Non-Annex 1	Asia-Pacific States	Lower-middle income	0.478	118	0.61	154	-0.94	-1
Namibia	Non-Annex 1	African States	Upper-middle income	0.44	106	1.74	121	0.56	0.05
Nauru	Non-Annex 1	Asia-Pacific States	High-income	NA	NA	6.56	40	0.43	-0.08
Nepal	Non-Annex 1	Asia-Pacific States	Lower-middle income	0.452	110	0.43	162	-0.09	-0.94
Netherlands	Annex 1	Western European and other States	High-income	0.043	4	8.77	21	1.53	1.85
New Zealand	Annex 1	Western European and other States	High-income	0.123	33	6.57	39	1.6	1.59
Nicaragua	Non-Annex 1	Latin American and Caribbean States	Lower-middle income	0.428	101	0.81	143	-1.1	-0.71
Niger	Non-Annex 1	African States	Low-income	0.642	154	0.10	185	-0.52	-0.62
Nigeria	Non-Annex 1	African States	Lower-middle income	NA	NA	0.67	149	-0.59	-1.03
Niue	Non-Annex 1	Asia-Pacific States	NA	NA	NA	NA	NA	1.16	NA
North Korea	Non-Annex 1	Asia-Pacific States	Low-income	NA	NA	0.71	145	-2.16	-1.46
North Macedonia	Non-Annex 1	Eastern European States	Upper-middle income	0.143	37	3.55	82	-0.06	0.14
Norway	Annex 1	Western European and other States	High-income	0.045	6	7.03	34	1.73	1.94
Oman	Non-Annex 1	Asia-Pacific States	High-income	0.306	68	15.19	12	-1.15	0.14
Pakistan	Non-Annex 1	Asia-Pacific States	Lower-middle income	0.538	135	0.98	136	-0.88	-0.55
Palau	Non-Annex 1	Asia-Pacific States	High-income	NA	NA	16.19	6	1.08	-0.05
Palestine	NA	Western European and other States	NA	NA	NA	NA	NA	NA	NA
Panama	Non-Annex 1	Latin American and Caribbean States	Upper-middle income	0.407	94	2.43	102	0.57	0.07

Papua New Guinea	Non-Annex 1	Asia-Pacific States	Lower-middle income	0.725	161	0.87	141	0.03	-0.85
Paraguay	Non-Annex 1	Latin American and Caribbean States	Upper-middle income	0.446	107	1.21	130	0.07	-0.47
Peru	Non-Annex 1	Latin American and Caribbean States	Upper-middle income	0.395	87	1.70	122	0.22	-0.24
Philippines	Non-Annex 1	Asia-Pacific States	Lower-middle income	0.43	104	1.33	128	-0.1	0.06
Poland	Annex 1	Eastern European States	High-income	0.115	28	8.24	25	0.62	0.38
Portugal	Annex 1	Western European and other States	High-income	0.075	17	4.84	60	1.26	1.02
Qatar	Non-Annex 1	Asia-Pacific States	High-income	0.185	43	32.42	1	-1.29	0.91
Republic of Moldova	Non-Annex 1	Eastern European States	Upper-middle income	0.204	46	3.17	90	-0.05	-0.46
Republic of the Congo	Non-Annex 1	African States	Lower-middle income	0.57	144	0.61	153	-1.29	-1.43
Romania	Annex 1	Eastern European States	Upper-middle income	0.276	61	3.85	74	0.58	-0.22
Russian Federation	Annex 1	Eastern European States	Upper-middle income	0.225	50	11.13	18	-1.08	0.03
Rwanda	Non-Annex 1	African States	Low-income	0.402	92	0.09	186	-1.1	0.34
Saint Kitts and Nevis	Non-Annex 1	Latin American and Caribbean States	High-income	NA	NA	4.96	58	0.82	0.7
Saint Lucia	Non-Annex 1	Latin American and Caribbean States	Upper-middle income	0.401	90	2.14	109	0.88	0.15
Saint Vincent and the Grenadines	Non-Annex 1	Latin American and Caribbean States	Upper-middle income	NA	NA	2.54	97	0.91	0.15
Samoa	Non-Annex 1	Asia-Pacific States	Lower-middle income	0.36	81	1.63	125	0.81	0.52
San Marino	Non-Annex 1	Western European and other States	High-income	NA	NA	NA	NA	1.12	NA
Sao Tome and Principe	Non-Annex 1	African States	Lower-middle income	0.537	133	0.66	150	0.35	-0.64
Saudi Arabia	Non-Annex 1	Asia-Pacific States	High-income	0.252	56	15.27	10	-1.61	0.15
Senegal	Non-Annex 1	African States	Lower-middle income	0.533	130	0.62	151	0.24	0.01

Serbia	Non-Annex 1	Eastern European States	Upper-middle income	0.132	35	6.52	41	-0.12	0.03
Serbia and Montenegro	NA	Eastern European States	NA	NA	NA	NA	NA	NA	NA
Seychelles	Non-Annex 1	African States	High-income	NA	NA	6.41	42	0.45	0.51
Sierra Leone	Non-Annex 1	African States	Low-income	0.644	155	0.13	182	-0.09	-1.02
Singapore	Non-Annex 1	Asia-Pacific States	High-income	0.065	12	8.40	24	-0.2	2.34
Slovakia	Annex 1	Eastern European States	High-income	0.191	45	6.06	47	0.88	0.54
Slovenia	Annex 1	Eastern European States	High-income	0.063	10	6.77	37	0.94	1.17
Solomon Islands	Non-Annex 1	Asia-Pacific States	Lower-middle income	NA	NA	0.57	155	0.55	-0.91
Somalia	Non-Annex 1	African States	Low-income	NA	NA	0.05	191	-1.8	-2.09
South Africa	Non-Annex 1	African States	Upper-middle income	0.406	93	7.50	31	0.7	0.3
South Korea	Non-Annex 1	Asia-Pacific States	High-income	0.064	11	12.23	15	0.82	1.42
South Sudan	Non-Annex 1	African States	Low-income	NA	NA	0.13	184	-1.83	-2.34
Spain	Annex 1	Western European and other States	High-income	0.07	16	5.52	52	1.01	0.89
Sri Lanka	Non-Annex 1	Asia-Pacific States	Lower-middle income	0.401	90	1.00	135	-0.05	-0.07
Sudan	Non-Annex 1	African States	Low-income	0.545	138	0.48	160	-1.43	-1.49
Suriname	Non-Annex 1	Latin American and Caribbean States	Upper-middle income	0.436	105	3.61	80	0.42	-0.54
Sweden	Annex 1	Western European and other States	High-income	0.039	3	3.54	83	1.5	1.72
Switzerland	Annex 1	Western European and other States	High-income	0.025	1	4.40	65	1.54	2.02
Syrian Arab Republic	Non-Annex 1	Asia-Pacific States	Low-income	0.482	122	1.65	124	-1.93	-1.73
Tajikistan	Non-Annex 1	Asia-Pacific States	Lower-middle income	0.314	70	0.81	144	-1.78	-0.71

Thailand	Non-Annex 1	Asia-Pacific States	Upper-middle income	0.359	80	3.71	76	-0.81	0.3
Timor-Leste	Non-Annex 1	Asia-Pacific States	Lower-middle income	NA	NA	0.50	159	0.37	-0.8
Togo	Non-Annex 1	African States	Low-income	0.573	145	0.29	170	-0.75	-0.69
Tonga	Non-Annex 1	Asia-Pacific States	Upper-middle income	0.354	79	1.84	116	0.64	0.16
Trinidad and Tobago	Non-Annex 1	Latin American and Caribbean States	High-income	0.323	73	12.78	13	0.64	0.18
Tunisia	Non-Annex 1	African States	Lower-middle income	0.296	65	2.59	96	0.27	-0.2
Türkiye	Annex 1	Asia-Pacific States	Upper-middle income	0.306	68	5.02	57	-0.86	-0.04
Turkmenistan	Non-Annex 1	Asia-Pacific States	Upper-middle income	NA	NA	12.26	14	-2.03	-1.16
Tuvalu	Non-Annex 1	Asia-Pacific States	Upper-middle income	NA	NA	0.87	140	1.18	-0.65
Uganda	Non-Annex 1	African States	Low-income	0.535	131	0.14	181	-0.72	-0.58
Ukraine	Annex 1	Eastern European States	Lower-middle income	0.234	52	4.15	67	0.09	-0.36
United Arab Emirates	Non-Annex 1	Asia-Pacific States	High-income	0.079	18	20.80	3	-1.18	1.33
United Kingdom of Great Britain and Northern Ireland	Annex 1	Western European and other States		0.118	31	5.40	54	1.25	1.38
United Republic of Tanzania	Non-Annex 1	African States	Lower-middle income	0.556	140	0.21	177	-0.71	-0.77
United States of America	Annex 1	Western European and other States	High-income	0.204	46	15.24	11	0.87	1.32
Uruguay	Non-Annex 1	Latin American and Caribbean States	High-income	0.288	62	1.89	113	1.31	0.78
Uzbekistan	Non-Annex 1	Asia-Pacific States	Lower-middle income	0.288	62	3.40	84	-1.54	-0.51
Vanuatu	Non-Annex 1	Asia-Pacific States	Lower-middle income	NA	NA	0.62	152	0.61	-0.46
Venezuela (Bolivarian Republic of)	Non-Annex 1	Latin American and Caribbean States	NA	0.479	119	4.78	61	-1.51	-1.78
Viet Nam	Non-Annex 1	Asia-Pacific States	Lower-middle income	0.296	65	2.70	93	-1.38	0.2

Yemen	Non-Annex 1	Asia-Pacific States	Low-income	0.795	162	0.33	166	-1.77	-2.31
Yugoslavia	NA	Eastern European States	NA	NA	NA	NA	NA	NA	NA
Zambia	Non-Annex 1	African States	Lower-middle income	0.539	137	0.45	161	-0.43	-0.77
Zimbabwe	Non-Annex 1	African States	Lower-middle income	0.527	129	0.85	142	-1.12	-1.25

Supplement Table 5. Overall summary characteristics of collected data.

**Data characteristic**	
Total number of Party delegations	5,152
Total number of Party delegates	149,010
Time period	1995 to 2023
Range of GII 2019	0.03-0.80
**Number of Party delegates within each sub-group**	
Type of party to the Convention	
Annex 1	30.5% (45,396)
Non-Annex 1	0.3% (423)
Not applicable	69.3% (103,191)
United Nations (UN) region	
African	31.7% (47,177)
Asia-Pacific	26.1% (38,892)
Eastern European	6.7% (9,994)
Latin American & Caribbean	12.5% (18,686)
Western European & other	23% (34,261)
World Bank (WB) socio-economic group	
High-income	33.8% (50,414)
Upper-middle income	12.1% (18,042)
Lower-middle income	28.3% (42,099)
Low-income	23.1% (34,492)
Not applicable	2.7% (3,963)
Inferred gender delegate	
Women & gender minorities	31.6% (47,143)
Men	68.4% (101,862)
Unknown	0% (5)
Inferred gender diversity	
Majority women & gender minorities	8.8%% (453)
Majority men	80.7% (4,156)
Gender parity	10.5% (543)

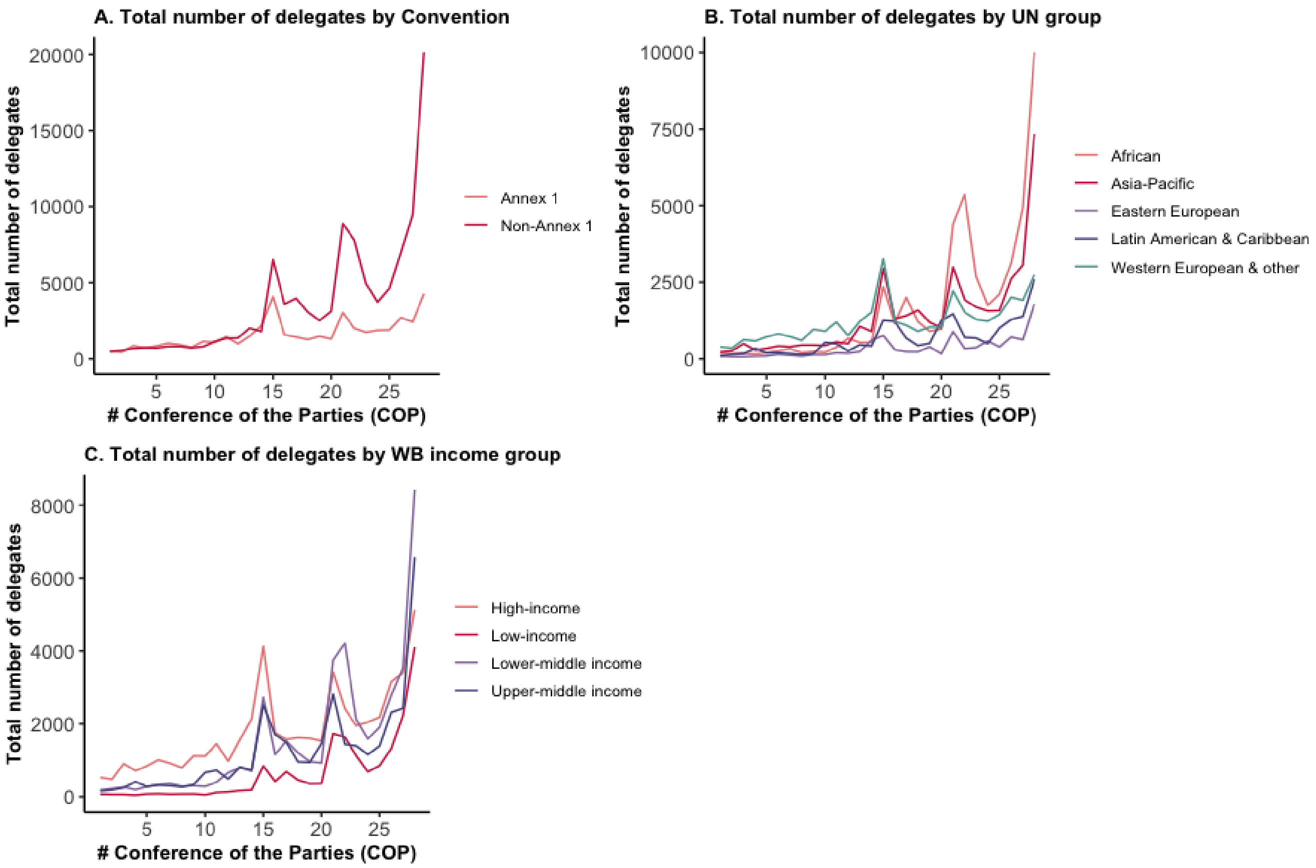

Supplement Figure 1. Total number of Parties' delegation members at each COP1-28 (1994-2023). Groupings provided by **A.** type of party to the Convention, **B.** United Nations (UN) region, and **C.** World Bank (WB) income group.

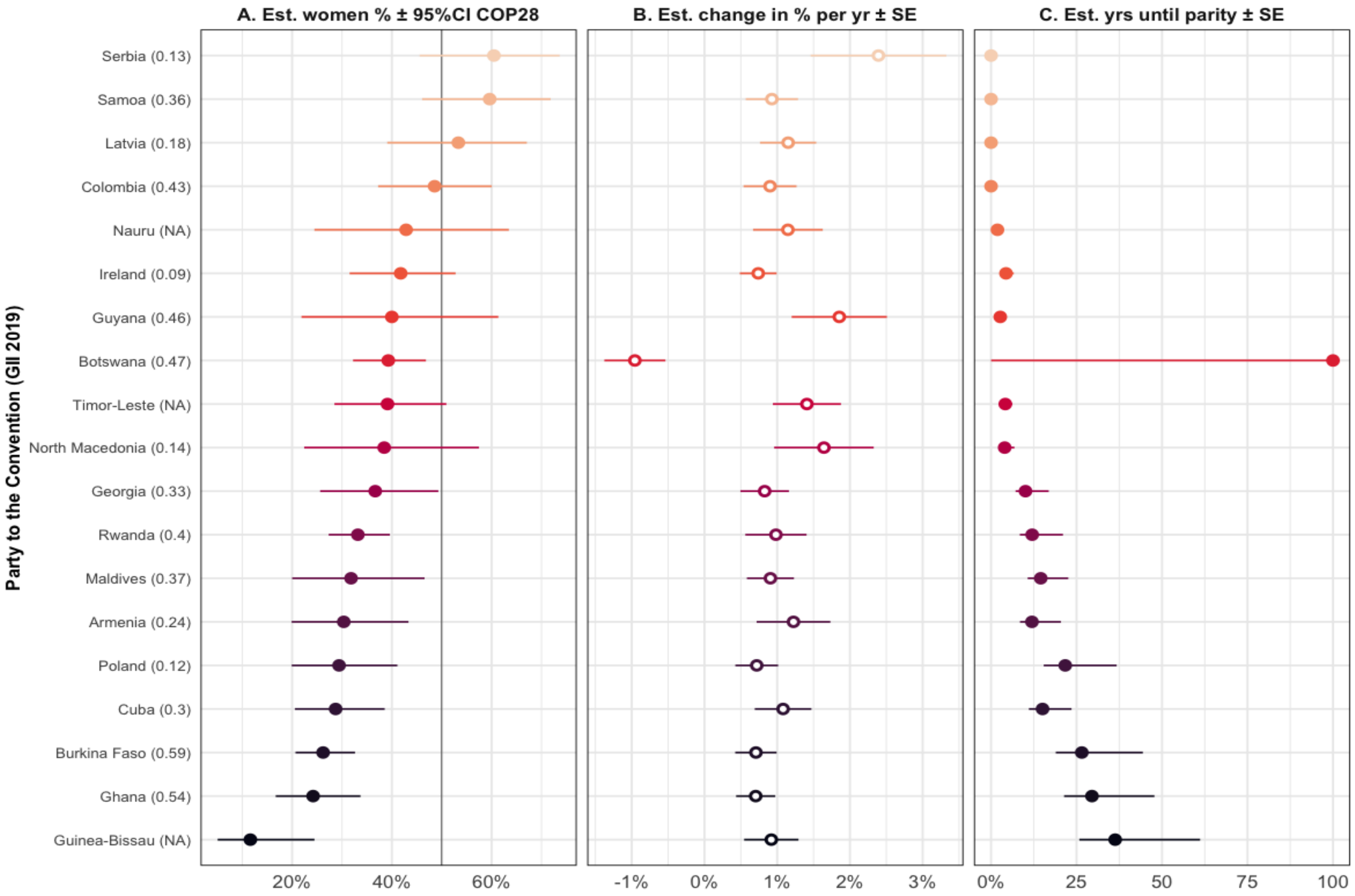

Supplement Figure 2. Women representation in Party delegations presented for those with a trend in estimated change of percentage of women delegation members per year, adjusted *P*-value < 0.05 but > 0.01. The Parties' Gender Inequality Index (GII) 2019 is depicted between brackets. **A.** Proportion (%) ± 95% confidence interval (CI) of inferred women delegation members at the COP27 (2022). **B.** Estimated change (%) ± standard error (SE) of inferred women delegation members at the COP per year. **C.** Estimated years ± SE until gender parity (45%–55% inferred women and gender minorities) from COP28 (2023). Note, only countries or territories that were a Party or Observer to the Convention in COP28 were included.

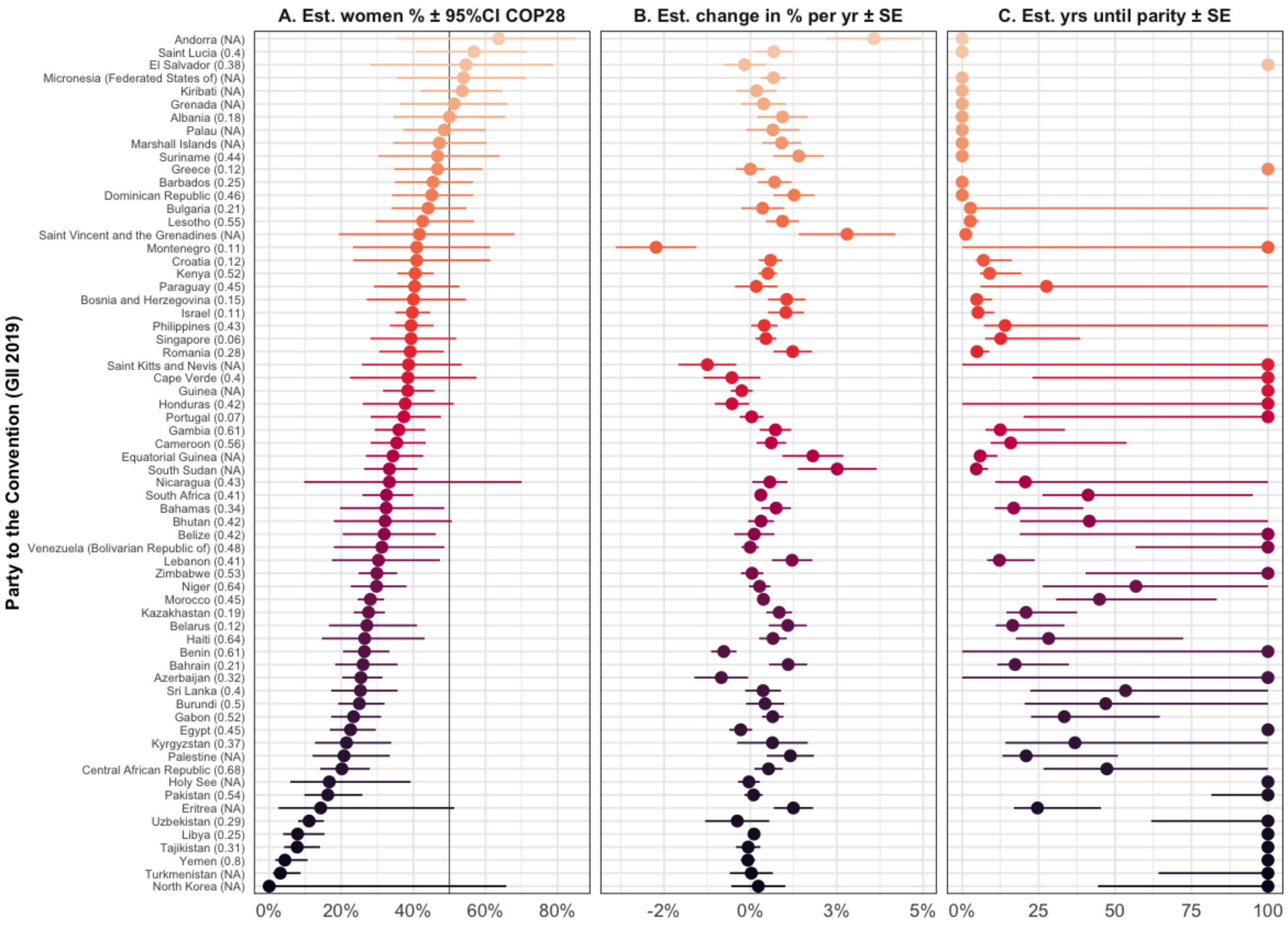

Supplement Figure 3. Women representation in Party delegations presented for those with a trend in estimated change of percentage of women delegation members per year, adjusted *P*-value > 0.05. The Parties' Gender Inequality Index (GII) 2019 is depicted between brackets. **A.** Proportion (%) ± 95% confidence interval (CI) of inferred women delegation members at the COP28 (2023). **B.** Estimated change (%) ± standard error (SE) of inferred women delegation members at the COP per year. **C.** Estimated years ± SE until gender parity (45%–55% inferred women and gender minorities) from COP28 (2023). Note, only countries or territories that were a Party or Observer to the Convention in COP28 were included.

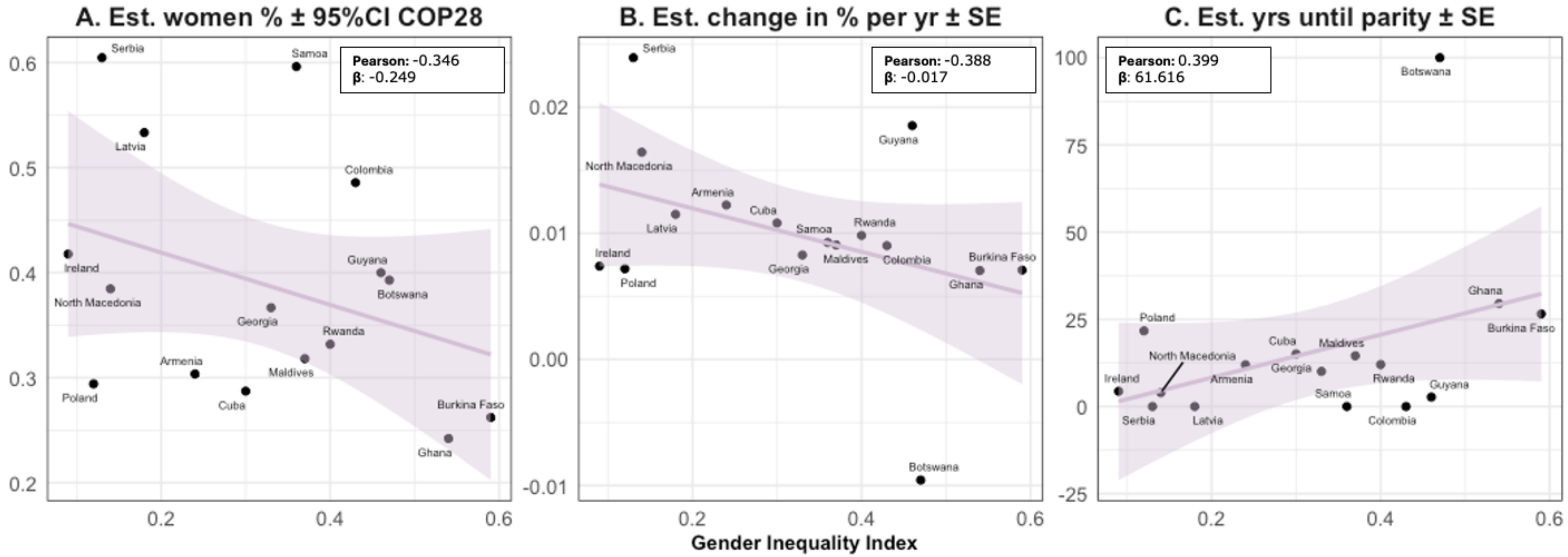

Supplement Figure 4. Women representation by Party Voice & Accountability Worldwide Governance Indicator (2019). A. Proportion (%) ± 95% Confidence Interval (CI) of inferred women delegation members at the COP28 (2023) by Voice & Accountability indicator 2019. **B.** Estimated change (%) ± standard error (SE) of inferred women delegation members at the COP per year by Voice & Accountability indicator 2019. **C.** Estimated years ± standard error (SE) until gender parity (45%-55% inferred women and gender minorities) from COP28 (2023) by Voice & Accountability indicator 2019. Note, only countries with a trend in estimated change of % women delegation members per year adjusted *P*-value < 0.05 but > 0.01were included. The Voice and Accountability Worldwide governance indicator is a reflection of the perceived extent to which a country's citizens are able to participate in selecting their government, freedom of expression, freedom of association and free media. Estimates of governance performance on these indicators ranges from 2.5 (strong) to -2.5 (weak)

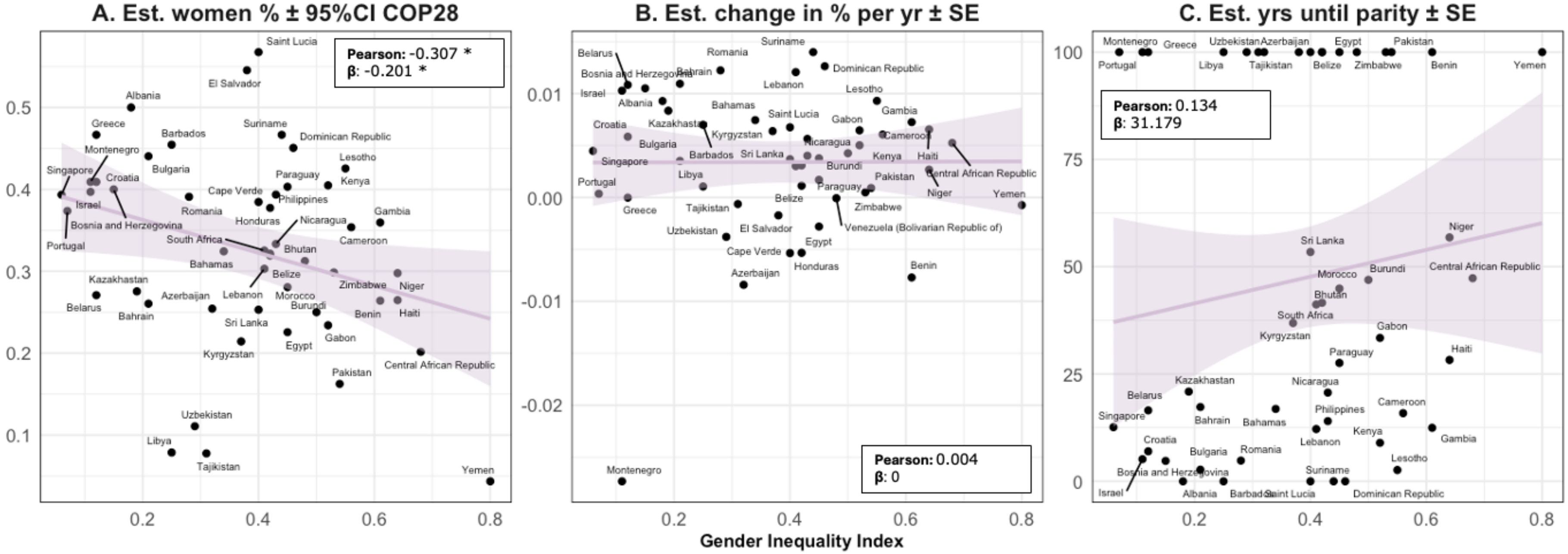

Supplement Figure 5. Women representation by Party Government Effectiveness Worldwide Governance Indicator (2019). A. Proportion (%) ± 95% Confidence Interval (CI) of inferred women delegation members at the COP28 by Government Effectiveness indicator 2019. **B.** Estimated change (%) ± standard error (SE) of inferred women delegation members at the COP per year by Government Effectiveness indicator 2019. **C.** Estimated years ± standard error (SE) until gender parity (45%-55% inferred women and gender minorities) from 2010-2019 by Government Effectiveness indicator 2019. Note, only countries with a trend in estimated change of % women delegation members per year adjusted P-value >0.05 were included. The Government Effectiveness Worldwide governance indicator reflects the perceived public services quality, civil service quality and degree of independence from political pressure, policy formulation and implementation quality, and the credibility of government's commitment to policies. Estimates of governance performance on these indicators range from 2.5 (strong) to -2.5 (weak).

References

1 van Daalen KR, Chowdhury M, Dada S, *et al.* Does global health governance walk the talk? Gender representation in World Health Assemblies, 1948–2021. BMJ Global Health. 2022; **7**: e009312.

2 Andersen JP, Schneider JW, Jagsi R, Nielsen MW. Gender variations in citation distributions in medicine are very small and due to self-citation and journal prestige. *Elife* 2019; **8**. DOI:10.7554/eLife.45374.

3 Bhatia S, Cotton CC, Kim E, *et al.* Gender and Nationality Trends in Manuscripts Published in Prominent Gastroenterology Journals Between 1997 and 2017. *Dig Dis Sci* 2022; **67**: 367–76.

4 Nielsen MW, Andersen JP, Schiebinger L, Schneider JW. One and a half million medical papers reveal a link between author gender and attention to gender and sex analysis. *Nat Hum Behav* 2017; **1**: 791–6.

5 United Nations Development Programme. Human development reports: gender inequality index (GII). https://hdr.undp.org/data-center/thematic-composite-indices/gender-inequality-index#/indicies/GII. 2019.

6 United Nations Framework Convention on Climate Change. UNFCCC Processes - Parties & Observers. https://unfccc.int/parties-observers (accessed Feb 10, 2022).

7 Thomas MA. What Do the Worldwide Governance Indicators Measure? *The European Journal of Development Research* 2010; **22**: 31–54.
